# Bologna Guidelines for Diagnosis and Management of Adhesive Small Bowel Obstruction (ASBO): 2010 Evidence-Based Guidelines of the World Society of Emergency Surgery

**DOI:** 10.1186/1749-7922-6-5

**Published:** 2011-01-21

**Authors:** Fausto Catena, Salomone Di Saverio, Michael D Kelly, Walter L Biffl, Luca Ansaloni, Vincenzo Mandalà, George C Velmahos, Massimo Sartelli, Gregorio Tugnoli, Massimo Lupo, Stefano Mandalà, Antonio D Pinna, Paul H Sugarbaker, Harry Van Goor, Ernest E Moore, Johannes Jeekel

**Affiliations:** 1 Emergency Surgery Unit, Department of General and Multivisceral Transplant Surgery, S. Orsola Malpighi University Hospital, Bologna, Italy; 2 Emergency and Trauma Surgery Unit, Departments of Emergency and Surgery, Maggiore Hospital Trauma Center, Bologna, Italy; 3 Upper GI Unit, Department of Surgery, Frenchay Hospital, North Bristol, NHS Trust, Bristol, UK; 4 Department of Surgery, Denver Health Medical Center/University of Colorado-Denver, Denver, Colorado 80204-4507, USA; 5 General Surgery I, Ospedali Riuniti di Bergamo, Bergamo, Italy; 6 Department of General and Emergency Surgery, Associated Hospitals "Villa Sofia - Cervello", Palermo, Italy; 7 Department of Surgery, Massachusetts General Hospital, Harvard Medical School, Boston, MA, USA; 8 Department of Surgery, Macerata Hospital - Via Santa Lucia 2, 62100 Macerata - Italy; 9 Washington Cancer Institute, Washington Hospital Center, Washington, DC 20010, USA; 10 Department of Surgery, Radboud University Nijmegen Medical Centre, P.O. Box 9101, 6500 HB, Nijmegen, The Netherlands; 11 Department of Surgery, Denver Health, University of Colorado Health Sciences Denver, Denver Health Medical Center, 777 Bannock Street, Denver, CO 80204, USA; 12 Department of Surgery, Erasmus University Medical Center, PO Box 2040, 3000 CA Rotterdam, The Netherlands

## Abstract

**Background:**

There is no consensus on diagnosis and management of ASBO. Initial conservative management is usually safe, however proper timing for discontinuing non operative treatment is still controversial. Open surgery or laparoscopy are used without standardized indications.

**Methods:**

A panel of 13 international experts with interest and background in ASBO and peritoneal diseases, participated in a consensus conference during the 1^st ^International Congress of the World Society of Emergency Surgery and 9^th ^Peritoneum and Surgery Society meeting, in Bologna, July 1-3, 2010, for developing evidence-based recommendations for diagnosis and management of ASBO. Whenever was a lack of high-level evidence, the working group formulated guidelines by obtaining consensus.

**Recommendations:**

In absence of signs of strangulation and history of persistent vomiting or combined CT scan signs (free fluid, mesenteric oedema, small bowel faeces sign, devascularized bowel) patients with partial ASBO can be managed safely with NOM and tube decompression (either with long or NG) should be attempted. These patients are good candidates for Water Soluble Contrast Medium (WSCM) with both diagnostic and therapeutic purposes. The appearance of water-soluble contrast in the colon on X-ray within 24 hours from administration predicts resolution. WSCM may be administered either orally or via NGT (50-150 ml) both immediately at admission or after an initial attempt of conservative treatment of 48 hours. The use of WSCM for ASBO is safe and reduces need for surgery, time to resolution and hospital stay.

NOM, in absence of signs of strangulation or peritonitis, can be prolonged up to 72 hours. After 72 hours of NOM without resolution surgery is recommended.

Patients treated non-operatively have shorter hospital stay, but higher recurrence rate and shorter time to re-admission, although the risk of new surgically treated episodes of ASBO is unchanged. Risk factors for recurrences are age <40 years and matted adhesions. WSCM does not affect recurrence rates or recurrences needing surgery when compared to traditional conservative treatment.

Open surgery is the preferred method for surgical treatment of strangulating ASBO as well as after failed conservative management. In selected patients and with appropriate skills, laparoscopic approach can be attempted using open access technique. Access in the left upper quadrant should be safe. Laparoscopic adhesiolysis should be attempted preferably in case of first episode of SBO and/or anticipated single band. A low threshold for open conversion should be maintained.

Peritoneal adhesions should be prevented. Hyaluronic acid-carboxycellulose membrane and icodextrin can reduce incidence of adhesions. Icodextrin may reduce the risk of re-obstruction. HA cannot reduce need of surgery.

## Introduction

Any intra-abdominal surgical procedure is a procedure inside the peritoneal organ. Intra-abdominal adhesions are strands or membranes of fibrous tissue that can be attached to the various intraabdominal organs, gluing them strongly together.

Abdominal adhesions, which can begin forming within a few hours after an operation, represent the most common cause of intestinal obstruction being responsible for 60% to 70% of SBO [1,2].

Complications of adhesions include chronic pelvic pain (20-50% incidence), small bowel obstruction (49-74% incidence), intestinal obstruction in ovarian cancer patients (22% incidence), and infertility due to complications in the fallopian tube, ovary, and uterus (15-20% incidence) [3,4]. Pelvic adhesions were found to be responsible in 15% to 40% of infertilities [5,6].

Intraabdominal adhesions related to prior abdominal surgery is the etiologic factor in up to 75% of cases of small-bowel obstruction. More than 300,000 patients are estimated to undergo surgery to treat adhesion-induced small-bowel obstruction in the United States annually. In details adhesiolysis was responsible for 303,836 hospitalizations during 1994, primarily for procedures on the digestive and female reproductive systems and these procedures accounted for 846,415 days of inpatient care and $1.3 billion in hospitalization and surgeon expenditures [7]. Foster et al. reported in 2005 that during the year 1997 in the state of California, SBO accounted for 32,583 unscheduled admissions, and approximately 85% were secondary to adhesions [8].

Abdominal adhesions pose a significant health problem with major adverse effects on quality of life, use of health care resources, and financial costs. Incidence rates for abdominal adhesions have been estimated to be as high as 94% [9] -95% [10] after laparotomies. The presence of adhesions makes re-operation more difficult, adds an average of 24 minutes to the surgery, increases the risk of iatrogenic bowel injury, and makes future laparoscopic surgery more difficult or even not possible [11,12].

## Background of Bologna Guidelines

Adhesive small bowel obstruction require appropriate management with a proper diagnostic and therapeutic pathway. Indication and length of Non Operative treatment and appropriate timing for surgery may represent an insidious issue.

Delay in surgical treatment may cause a substantial increase of morbidity and mortality. However repeated laparotomy and adhesiolysis may worsen the process of adhesion formation and their severity. Furthermore the introduction and widespread of laparoscopy has raised the question of selection of appropriate patients with ASBO good candidate for laparoscopic approach. On the other hand, several adjuncts for improving the success rate of NOM and clarifying indications and timing for surgery are currently available, such as hyperosmolar water soluble contrast medium.

No consensus has been reached in diagnosing and managing the patients with ASBO and specific and updated guidelines are lacking.

We carried out an extensive review of the English-language literature and found that there was little high-level evidence in this field, and no systematically described practical manual for the field. Most importantly, there are no standardized diagnostic criteria and therapeutic management guidelines for ASBO, therefore, we would like to establish standards for these items. The Bologna Guidelines include evidence-based medicine and reflect the international consensus obtained through earnest discussions among professionals in the field on 1-3 July, 2010, at the Belmeloro Convention Center, Bologna, Italy.

### Notes on the use of the Guidelines

The Guidelines are evidence-based, with the grade of recommendation also based on the evidence. The Guidelines present the diagnostic and therapeutic methods for optimal management and prevention of ASBO.

The practice Guidelines promulgated in this work do not represent a standard of practice. They are suggested plans of care, based on best available evidence and the consensus of experts, but they do not exclude other approaches as being within the standard of practice. For example, they should not be used to compel adherence to a given method of medical management, which method should be finally determined after taking account of the conditions at the relevant medical institution (staff levels, experience, equipment, etc.) and the characteristics of the individual patient. However, responsibility for the results of treatment rests with those who are directly engaged therein, and not with the consensus group.

## Methods

### - Consensus Development

In the Consensus Conference on July 2^nd ^2010, the expert panel had two meetings and a further plenary session. The aim was to focus and clarify the diagnostic and therapeutic issues of the complex management of ASBO, leading to new clinical guidelines, updated and including a wide range of recommendations, for diagnosis, non operative management, timing for surgery, type of surgery and prevention strategies of peritoneal post-operative adhesions causing small bowel obstruction. Based on the review of the current literature, a panel of worldwide experts were invited to participate in the development of the new guidelines. All members of the expert panel were asked to define ASBO. For each step of diagnosis, treatment (conservative and surgical) and prevention of ASBO, one expert summarized the current state of the art. From the evidence based presentations and the reported statements as well as from the results of the relevant literature review, a preliminary document with the resume of the Consensus Statements and Recommendations was compiled. For every key statement, the discussion within the expert panel with the involvement of the audience, took place until a 100% consensus within the group and the audience was achieved. Comments from the audience were collected and partly included in the manuscript. In September 2010 the expert panel had further contacts for discussing and finalize the final version of the text of the guidelines recommendations. The final version of the guidelines was approved by all experts in the panel as well as the experts from the audience who played an active role in the discussion during the Consensus Conference. Each ''chapter'' consists of a key statement with a grade of recommendation (GoR) followed by a commentary to explain the rationale and evidence behind the statement.

#### - Literature Searches and Appraisal

We have used the Oxford hierarchy for grading clinical studies according to levels of evidence. Literature searches were aimed at finding randomized (i.e., level 1b evidence) or nonrandomized controlled clinical trials (i.e., level 2b evidence). Alternatively, low-level evidence (mainly case series and case reports; i.e., level 4 evidence) was reviewed. Studies containing severe methodological flaws were downgraded. For each intervention, we considered the validity and homogeneity of study results, effect sizes, safety, and economic consequences.

Systematic literature searches were conducted on Medline and the Cochrane Library until December 2010. There were no restrictions regarding the language of publication. We also paid attention to studies that were referenced in systematic reviews or previous guidelines [http://www.east.org/tpg/sbo.pdf] [13].

#### - Categories of Evidence and Grades of Recommendation

All studies have been evaluated for quality according to STARD checklist for the reporting of studies of diagnostic accuracy (http://www.stard-statement.org)(Table 1). Categories of evidence and Grades of Recommendation have been assessed and classified according to the Oxford Centre for Evidence-based Medicine Levels of Evidence (Version March 2009) (Table 2 and Table 3).

**Table 1:** STARD checklist for the reporting of studies of diagnostic accuracy

Section and Topic	Item #	
TITLE/ABSTRACT/KEYWORDS	1	Identify the article as a study of diagnostic accuracy (recommended MeSH heading 'sensitivity and specificity')

INTRODUCTION	2	State the research questions or study aims, such as estimating diagnostic accuracy or comparing accuracy between tests or across participant groups

METHODS		

*Participants*	3	Describe the study population: The inclusion and exclusion criteria, setting and locations where the data were collected

	4	Describe participant recruitment: Was recruitment based on presenting symptoms, results from previous tests, or the fact that the participants had received the index tests or the reference standard?

	5	Describe participant sampling: Was the study population a consecutive series of participants defined by the selection criteria in items 3 and 4? If not, specify how participants were further selected

*Test methods*	6	Describe data collection: Was data collection planned before the index test and reference standard were performed (prospective study) or after (retrospective study)?

	7	Describe the reference standard and its rationale

	8	Describe technical specifications of material and methods involved including how and when measurements were taken, and/or cite references for index tests and reference standard

	9	Describe definition of and rationale for the units, cutoffs and/or categories of the results of the index tests and the reference standard

	10	Describe the number, training and expertise of the persons executing and reading the index tests and the reference standard

*Statistical methods*	11	Describe whether or not the readers of the index tests and reference standard were blind (masked) to the results of the other test and describe any other clinical information available to the readers

	12	Describe methods for calculating or comparing measures of diagnostic accuracy, and the statistical methods used to quantify uncertainty (e.g. 95% confidence intervals)

RESULTS	13	Describe methods for calculating test reproducibility, if done

*Participants*		

	14	Report when study was done, including beginning and ending dates of recruitment

	15	Report clinical and demographic characteristics of the study population (e.g. age, sex, spectrum of presenting symptoms, comorbidity, current treatments, recruitment centers

*Test results*	16	Report the number of participants satisfying the criteria for inclusion that did or did not undergo the index tests and/or the reference standard; describe why participants failed to receive either test (a flow diagram is strongly recommended)

	17	Report time interval from the index tests to the reference standard, and any treatment administered between

	18	Report distribution of severity of disease (define criteria) in those with the target condition; other diagnoses in participants without the target condition

	19	Report a cross tabulation of the results of the index tests (including indeterminate and missing results) by the results of the reference standard; for continuous results, the distribution of the test results by the results of the reference standard

*Estimates*	20	Report any adverse events from performing the index tests or the reference standard

	21	Report estimates of diagnostic accuracy and measures of statistical uncertainty (e.g. 95% confidence intervals)

	22	Report how indeterminate results, missing responses and outliers of the index tests were handled.

	23	Report estimates of variability of diagnostic accuracy between subgroups of participants, readers or centers, if done.

DISCUSSION	24	Report estimates of test reproducibility, if done
	25	Discuss the clinical applicability of the study findings

MeSH: Medical subject heading

STARD: STAndards for the **Reporting **of Diagnostic **accuracy **studies

*This checklist is found at:*http://www.consort-statement.org/index.aspx?o=2965  and  http://www.consort-statement.org/index.aspx?o=2967

**Table 2:** Categories of evidence (refer to levels of evidence and grades of recommendations on the homepage of the Centre for Evidence-Based Medicine) http://www.cebm.net/index.aspx?o=1025 Oxford Centre for Evidence-based Medicine Levels of Evidence (March 2009) (for definitions of terms used see glossary at http://www.cebm.net/?o=1116)

Level	Therapy/Prevention, Aetiology/Harm	Prognosis	Diagnosis	Differential diagnosis/symptom prevalence study	Economic and decision analyses
1a	SR (with homogeneity*) of RCTs	SR (with homogeneity*) of inception cohort studies; CDR† validated in different populations	SR (with homogeneity*) of Level 1 diagnostic studies; CDR† with 1b studies from different clinical centres	SR (with homogeneity*) of prospective cohort studies	SR (with homogeneity*) of Level 1 economic studies
1b	Individual RCT (with narrow Confidence Interval‡)	Individual inception cohort study with > 80% follow-up; CDR† validated in a single population	Validating** cohort study with good††† reference standards; or CDR† tested within one clinical centre	Prospective cohort study with good follow-up****	Analysis based on clinically sensible costs or alternatives; systematic review(s) of the evidence; and including multi-way sensitivity analyses
1c	All or none§	All or none case-series	Absolute SpPins and SnNouts††	All or none case-series	Absolute better-value or worse-value analyses ††††
2a	SR (with homogeneity*) of cohort studies	SR (with homogeneity*) of either retrospective cohort studies or untreated control groups in RCTs	SR (with homogeneity*) of Level >2 diagnostic studies	SR (with homogeneity*) of 2b and better studies	SR (with homogeneity*) of Level >2 economic studies
2b	Individual cohort study (including low quality RCT; e.g., <80% follow-up)	Retrospective cohort study or follow-up of untreated control patients in an RCT; Derivation of CDR† or validated on split-sample§§§ only	Exploratory** cohort study with good††† reference standards; CDR† after derivation, or validated only on split-sample§§§ or databases	Retrospective cohort study, or poor follow-up	Analysis based on clinically sensible costs or alternatives; limited review(s) of the evidence, or single studies; and including multi-way sensitivity analyses
2c	"Outcomes" Research; Ecological studies	"Outcomes" Research		Ecological studies	Audit or outcomes research
3a	SR (with homogeneity*) of case-control studies		SR (with homogeneity*) of 3b and better studies	SR (with homogeneity*) of 3b and better studies	SR (with homogeneity*) of 3b and better studies
3b	Individual Case-Control Study		Non-consecutive study; or without consistently applied reference standards	Non-consecutive cohort study, or very limited population	Analysis based on limited alternatives or costs, poor quality estimates of data, but including sensitivity analyses incorporating clinically sensible variations.
4	Case-series (and poor quality cohort and case-control studies§§)	Case-series (and poor quality prognostic cohort studies***)	Case-control study, poor or non-independent reference standard	Case-series or superseded reference standards	Analysis with no sensitivity analysis
5	Expert opinion without explicit critical appraisal, or based on physiology, bench research or "first principles"	Expert opinion without explicit critical appraisal, or based on physiology, bench research or "first principles"	Expert opinion without explicit critical appraisal, or based on physiology, bench research or "first principles"	Expert opinion without explicit critical appraisal, or based on physiology, bench research or "first principles"	Expert opinion without explicit critical appraisal, or based on economic theory or "first principles"

Produced by Bob Phillips, Chris Ball, Dave Sackett, Doug Badenoch, Sharon Straus, Brian Haynes, Martin Dawes since November 1998. Updated by Jeremy Howick March 2009.

*Notes*

Users can add a minus-sign "-" to denote the level of that fails to provide a conclusive answer because:

***EITHER ***a single result with a wide Confidence Interval

***OR ***a Systematic Review with troublesome heterogeneity.

Such evidence is inconclusive, and therefore can only generate Grade D recommendations.

* By homogeneity we mean a systematic review that is free of worrisome variations (heterogeneity) in the directions and degrees of results between individual studies. Not all systematic reviews with statistically significant heterogeneity need be worrisome, and not all worrisome heterogeneity need be statistically significant. As noted above, studies displaying worrisome heterogeneity should be tagged with a "-" at the end of their designated level.

† Clinical Decision Rule. (These are algorithms or scoring systems that lead to a prognostic estimation or a diagnostic category.)

‡ See note above for advice on how to understand, rate and use trials or other studies with wide confidence intervals.

§ Met when all patients died before the Rx became available, but some now survive on it; or when some patients died before the Rx became available, but none now die on it.

§§ By poor quality cohort study we mean one that failed to clearly define comparison groups and/or failed to measure exposures and outcomes in the same (preferably blinded), objective way in both exposed and non-exposed individuals and/or failed to identify or appropriately control known confounders and/or failed to carry out a sufficiently long and complete follow-up of patients. By poor quality case-control study we mean one that failed to clearly define comparison groups and/or failed to measure exposures and outcomes in the same (preferably blinded), objective way in both cases and controls and/or failed to identify or appropriately control known confounders.

§§§ Split-sample validation is achieved by collecting all the information in a single tranche, then artificially dividing this into "derivation" and "validation" samples.

†† An "Absolute SpPin" is a diagnostic finding whose Specificity is so high that a Positive result rules-in the diagnosis. An "Absolute SnNout" is a diagnostic finding whose Sensitivity is so high that a Negative result rules-out the diagnosis.

‡‡ Good, better, bad and worse refer to the comparisons between treatments in terms of their clinical risks and benefits.

††† Good reference standards are independent of the test, and applied blindly or objectively to applied to all patients. Poor reference standards are haphazardly applied, but still independent of the test. Use of a non-independent reference standard (where the 'test' is included in the 'reference', or where the 'testing' affects the 'reference') implies a level 4 study.

†††† Better-value treatments are clearly as good but cheaper, or better at the same or reduced cost. Worse-value treatments are as good and more expensive, or worse and the equally or more expensive.

** Validating studies test the quality of a specific diagnostic test, based on prior evidence. An exploratory study collects information and trawls the data (e.g. using a regression analysis) to find which factors are 'significant'.

*** By poor quality prognostic cohort study we mean one in which sampling was biased in favour of patients who already had the target outcome, or the measurement of outcomes was accomplished in <80% of study patients, or outcomes were determined in an unblinded, non-objective way, or there was no correction for confounding factors.

**** Good follow-up in a differential diagnosis study is >80%, with adequate time for alternative diagnoses to emerge (for example 1-6 months acute, 1 - 5 years chronic)

**Table 3:** Grading system for ranking recommendations in clinical guidelines

Grade of recommendation	
A	Good evidence to support a recommendation for use
B	Moderate evidence to support a recommendation for use
C	Poor evidence to support a recommendation, or the effect may not exceed the adverse effects and/or inconvenience (toxicity, interaction between drugs and cost)
D	Moderate evidence to support a recommendation against use
E	Good evidence to support a recommendation against use

## Results

### - Definition, risk factors, natural history and diagnosis

Patients with ASBO treated nonsurgically have shorter hospital stay, however they have an higher recurrence rate, shorter time to re-admission, although the risk of new surgically treated episodes of ASBO is the same. (Level of Evidence 2b)

All patients being evaluated for small bowel obstruction should have plain films (Level of Evidence 2b GoR C)

CT-scans should not be routinely performed in the decision-making process except when clinical history, physical examination, and plain film are not conclusive for small bowel obstruction diagnosis (Level of Evidence 2b GoR B)

The association of CT scan signs of bowel ischemia should lead a low threshold for surgical intervention (Level of Evidence 2a GoR B)

MRI and US are of limited value for ASBO and should be limited to patients with contraindications for CT scan/iodine contrast (Level of Evidence 2c GoR C)

In patients undergoing initial non operative conservative management, a water-soluble contrast follow-through should be performed in order to rule out complete ASBO and predict the need for surgery (Level of Evidence 1b GoR A)

Adhesional postoperative small bowel obstruction is characterized by the presence of abdominal pain, vomiting, distention, and obstipation, in conjunction of confirmatory imaging.

SBO can be classified according to completeness: Partial vs. Complete (or high grade vs. low grade), according to etiology: Adhesional vs. Non-adhesional, according to timing: Early vs. Late (>30 days after surgery).

The most important risk factor for adhesive SBO is the type of surgery and extent of peritoneal damage. Surgeries of the colon and rectum are associated with a higher risk of adhesion-related problems [14]. Total colectomy with ileal pouch-anal anastomosis is the procedure with the highest incidence for adhesion-related problems with an overall incidence of SBO of 19.3%. Other high-risk procedures include gynecologic surgeries (11.1%) and open colectomy (9.5%).

Other possible risk factors include age younger than 60 years, previous laparotomy within 5 years, peritonitis, multiple laparotomies, emergency surgery, omental resection, and penetrating abdominal trauma, especially gunshot wounds [15-18].

The number of prior episodes is the strongest predictor of recurrence; in fact ASBO recurred after 53% of initial episodes and 85% or more of second, third, or later episodes in the experience of Barkan et al. Recurrence occurred sooner and more frequently in patients managed nonoperatively than in patients managed operatively [19].

With growing numbers of previous episodes of SBO requiring adhesiolysis, the risk for future re-admission for SBO increases, thus nonsurgical management of the initial episode has been advocated as a risk factor for recurrence [20].

Age younger than 40 years, the presence of matted adhesions, and surgical complications during the surgical management of the first episode as independent risks for recurrence [21].

Williams et al. [22] in a retrospective review of 329 patients (487 admissions) demonstrated that operatively treated patients had a lower frequency of recurrence (26.8% vs 40.5% *P *< 0.009) and a longer time interval to recurrence (411 *vs *153 days *P *< 0.004); however, they also had a longer hospital stay than that of patients treated nonoperatively (12.0 *vs *4.9 days; *P *< 0.0001). There was no significant difference in treatment type or in incidence or type of prior surgery among patients with early and late small bowel obstruction. The authors have also reported [23] early postoperative mortality of 3% and long-term mortality of 7% with the following independent risk factors: age >75 years old, medical complications, and a mixed mechanism of obstruction. Prevalence of medical and surgical morbidity was 8% and 6%, respectively. Independent risk factors for medical complications were ASA class >/= III and bands and for the surgical complications the number of obstructive structures >/= 10, a nonresected intestinal wall injury, and intestinal necrosis.

In a further multicenter prospective study [24] including 286 patients operated for ASBO and followed up for 41 months, cumulative incidence of overall recurrence was 15.9%, and for surgically managed recurrence 5.8%. The risk factors for the overall recurrences were age <40 years (hazard ratio [HR], 2.97), adhesion or matted adhesion (HR, 3.79) and, for the surgically managed: adhesions or matted adhesions (HR, 3.64), and postoperative surgical complications (HR, 5.63). In this study the number of recurring patients (21%) in absence of resection is very high. The beneficial effect of intestinal resection might relate to the decrease of the traumatized intestinal serosa area. In this way, it may be hypothesized that adhesive postoperative SBO frequency is linked to the extent of both the parietal peritoneal trauma (incision and site) and the intestinal serosa. Miller et al. [25] in a review of 410 patients accounting for 675 admissions found that a history of colorectal surgery and vertical incisions tended to predispose to multiple matted adhesions rather than an obstructive band. They conclude that the likelihood of reobstruction increases and the time to reobstruction decreases with increasing number of previous episodes of obstruction. Patients with matted adhesions have a greater recurrence rate than those with band adhesions. These authors failed to find reliable clinical indicators of impending strangulation and the optimum length of a non operative trial for patients with acute ASBO remains controversial.

Fevang et al. described the long term prognosis of 500 patients operated for ASBO with a median follow-up of 10 years and a maximum follow-up time of 40 years [26]. The cumulative recurrence rate for patients operated once for ASBO was 18% after 10 years and 29% at 30 years. For patients admitted several times for ASBO, the relative risk of recurrent ASBO increased with increasing number of prior ASBO episodes. The cumulative recurrence rate reached 81% for patients with 4 or more ASBO admissions. Other factors influencing the recurrence rate were the method of treatment of the last previous ASBO episode (conservative versus surgical) and the number of abdominal operations prior to the initial ASBO operation. The authors concluded that the risk of recurrence increased with increasing number of ASBO episodes. Most recurrent ASBO episodes occur within 5 years after the previous one, but a considerable risk is still present 10 to 20 years after an ASBO episode. Surgical treatment decreased the risk of future admissions for ASBO, but the risk of new surgically treated ASBO episodes was the same regardless of the method of treatment. Thus surgical treatment of a recurrent ASBO episode was associated with a significantly decreased risk of having conservatively treated ASBO episodes in the future, but the need for subsequent surgery for ASBO was similar regardless of the method of treatment.

First step of diagnostic work up [27] for ASBO is supine and erect plain abdominal X-ray. Radiologic stigmata of SBO are the presence/coincidence of multiple air-fluid levels, dilatation/distension of small bowel loops and the absence of gas in the colonic section. Plain film has sensitivity and specificity ranging from 65% to 80% [28]. Ultrasound can be useful only in expert hands; US is usually of limited value in bowel obstruction and/or in patients with distended bowel because the air, limiting ultrasound transmission, may obscure the underlying findings. The scan should be performed through flanks to avoid distended SB [29]. Usual US findings are: distention, peristalsis (differential diagnosis of ileus vs. mechanical SBO), differences in mucosal folds around transition point, free fluid (sign of ischemia) [30].

CT scan is highly diagnostic in SBO and has a great value in all patients with inconclusive plain films for complete or high grade SBO [31]. However CT-scans should not be routinely performed in the decision-making process except when clinical history, physical examination, and plain film are not conclusive for small bowel obstruction diagnosis [32]. CT can confirm the presence of complete obstruction and allow the diagnosis of the cause of SBO, it can also exclude a non-adhesional pathology and assess the occurrence of strangulation with a sensitivity and specificity higher than 90% and a NPV of nearly 100% [33].

#### IV contrast is necessary. Oral is not

Water-soluble contrast follow-through is valuable in patients undergoing initial non operative conservative management in order to rule out complete ASBO and predict the need for surgery [34].

This investigation is safer than barium in cases of perforation and peritoneal spread and has possible therapeutic value in the case of adhesive small intestine obstruction [35].

MRI use should be restricted to those patients having CT or iodine contrast contraindications.

### - Conservative treatment and timing for surgery

The management of small bowel obstruction caused by adhesions is controversial because surgery can induce new adhesions, whereas conservative treatment does not remove the cause of the obstruction [36]. Conservative treatment involves nasogastric intubation, intravenous fluid administration, and clinical observation. Strangulation of the bowel requires immediate surgery, but intestinal ischemia can be difficult to determine clinically. Several issues are raised when managing patients with ASBO.

#### The first question is whether to operate or not to operate

Patients without the signs of strangulation or peritonitis or history of persistent vomiting or combination of CT scan signs (free fluid, mesenteric oedema, lack of faeces signa, devascularized bowel) and partial ASBO can safely undergo non-operative management (LoE 1a GoR A)

In the absence of any signs of strangulation, patients with an adhesive SBO can be managed safely with non-operative treatment and tube decompression should be attempted (Level of Evidence 1b GoR A)

Patients who had surgery within the six weeks before the episode of small bowel obstruction, patients with signs of strangulation or peritonitis (fever, tachycardia and leucocytosis, metabolic acidosis and continuous pain), patients with irreducible hernia and patients who started to have signs of resolution at the time of admission are NOT candidate for conservative treatment +/- WSCA administration (Level of Evidence 1a GoR A)

Foster et al. in a population based appraisal [37] found that patients who underwent operations during index admission had longer lengths of stay, lower mortality, fewer SBO readmissions, and longer time to readmission than patients treated nonsurgically.

In a retrospective analysis of 123 patients admitted for ASBO and having an initial period of non-operative treatment, complete resolution occurred within 48 h in 75 (88%) cases, the remaining 10 had resolved by 72 h [38]. On the other hand only three (2.4%) patients, initially treated non-operatively, had small bowel strangulation. All three were operated on within 24 h of admission when changes in clinical findings suggested small bowel strangulation may be present. There were no deaths in the group having an initial period of non-operative treatment. Therefore, upon the authors conclusion, in the absence of any signs of strangulation, patients with an adhesive SBO can be managed safely with non-operative treatment.

In a prospective, randomized trial conducted to compare NGT and LT decompression with respect to the success of nonoperative treatment and morbidity of surgical intervention in 55 patients with acute ASBO, out of 28 patients managed with NGT and 27 with LT, twenty-one patients ultimately required operation [39]. At operation, 3 patients in the NGT group had ischemic bowel that required resection. Postoperative complications occurred in 23% of patients treated with NGT versus 38% of patients treated with LT and no deaths were observed. Therefore patients with ASBO can safely be given a trial of tube decompression upon hospital admission, given the absence of complications in patients treated with either type of tube decompression coupled with acceptable morbidity rate.

In patients with repeated episodes and many prior laparotomies for adhesions, prolonged conservative treatment, including parenteral nutritional support may be prudent and often avoid a complex high-risk procedure [40].

Fevang et al. found that among 146 patients with SBO initially treated conservatively, 93 (64%) settled without operation, 9 (6%) had strangulated bowel and 3 (2%) died [41]. Whereas of the 91 patients with partial obstruction but no sign of strangulation, 72 (79%) resolved on conservative treatment. Therefore the authors recommended that patients with partial obstruction and no sign of strangulation should initially be treated conservatively. Furthermore when complete obstruction is present, it may settle on conservative management, but the use of supplementary diagnostic tools might be desirable to find the patients who will need early operative treatment.

In another review, out of 329 patients with SBO 43% were successfully treated conservatively, whereas 57% failed conservative treatment and underwent surgery [42]. Overall, there were eight early deaths, four in each group (2.8% conservative vs. 2.1% surgical; p = ns). Out of these patients presenting with SBO, 63% had abdominal surgery and 37% had no prior abdominal surgery before developing a small bowel obstruction.

In conclusion, the most recent meta-analyses [43-45] showed that the patients who had surgery within the six weeks before the episode of small bowel obstruction, patients with signs of strangulation or peritonitis (fever, tachycardia and leucocytosis), patients with carcinomatosis, patients with irreducible hernia and patients who started to have signs of resolution at the time of admission are not candidate for conservative treatment +/- Water Soluble Contrast Medium administration.

Also the EAST guidelines on SBO management recommend that the patients with plain film finding of small bowel obstruction and Clinical markers (fever, leukocytosis, tachycardia, metabolic acidosis and continuous pain) or peritonitis on physical exam warrant exploration [46].

#### The second question is who can be safely managed with initial conservative management and which factors can reliably predict surgery

Complete SBO (no evidence of air within the large bowel) and increased serum creatine phosphokinase predicts NOM failure (Level of Evidence 2b GoR C)

Free intraperitoneal fluid, mesenteric edema, lack of the ''small bowel feces sign'' at CT, and history of vomiting, severe abdominal pain (VAS > 4), abdominal guarding, raised WCC and devascularized bowel at CT predict the need for emergent laparotomy at the time of admission (Level of Evidence 2c GoR C)

The appearance of water-soluble contrast in the colon on abdominal X ray within 24 hours of its administration predicts resolution of ASBO (Level of Evidence 1a GoR A)

Among patients with adhesive small bowel obstruction (ASBO) initially managed with a conservative strategy, predicting risk of operation is difficult.

Several recent studies have tried to focus on identifying predictive factors for failure of NOM and need for surgery.

In conservatively treated patients with ASBO, the drainage volume through the long tube on day 3 (cut-off value; 500mL) was the indicator for surgery [47].

In 2010 Komatsu et al. have developed a simple model for predicting the need of surgery in patients who initially undergo conservative management for ASBO. The model included 3 variables: age >65 years, presence of ascites on CT scan and drainage volume from NGT or LT > 500 mL on day 3. PPV of this model in the high-risk class was 72% with specificity of 96%, whereas NPV in the low risk class was 100% with sensitivity of 100% [48].

Tachycardia, fever, focal tenderness, increased white blood cell counts, and elevated lactate levels can indicate intestinal ischemia, but these indicators are not very specific [49]. When intestinal ischemia is unlikely, a conservative approach can be followed for 24-48 h. Meagher et al. have suggested that surgery is unavoidable in patients with small bowel obstruction after previous appendectomy or surgery on the fallopian tubes or ovaries [50].

In another recently developed model for predicting the risk of strangulated SBO, six variables correlated with small bowel resection: history of pain lasting 4 days or more, guarding, C-reactive protein level at least 75 mg/l, leucocyte count 10 × 10(9)/l or greater, free intraperitoneal fluid volume at least 500 ml on computed tomography (CT) and reduction of CT small bowel wall contrast enhancement [51].

A further multivariate predictive model of surgical operation in SBO [52], showed free intraperitoneal fluid, mesenteric edema, lack of the ''small bowel feces sign'' at CT, and history of vomiting to be significant predictors of the need for operative exploration.

In a retrospective study of 53 patients with ASBO treated using a long nasointestinal tube (LT), complete SBO (no evidence of air within the large bowel) and increased serum creatine phosphokinase (>or = 130 IU/L) were independent predictive factors for LT decompression failure [53].

A recent prospective study aimed to evaluate an algorithm using CT-scans and Gastrografin in the management of small bowel obstruction, severe abdominal pain (VAS > 4), abdominal guarding, raised WCC and devascularized bowel at CT predict the need for emergent laparotomy at the time of admission [54]. Furthermore this study demonstrated the diagnostic role of Gastrografin in discriminating between partial and complete small bowel obstruction whilst CT-scans were disappointing in their ability to predict the necessity of emergent laparotomies.

Again two systematic reviews confirmed the value of water soluble contrast medium in predicting need for surgery in ASBO patients.

Abbas et al. in 2007 already confirmed that Water-soluble contrast followed by an abdominal radiograph after at least 4 hours can accurately predict the likelihood of resolution of a small bowel obstruction [55] and that appearance of water-soluble contrast agent in the colon on an abdominal radiograph within 24 h of its administration predicted resolution of obstruction with a pooled sensitivity of 97 per cent and specificity of 96 per cent [56].

Branco et al. as well found that the appearance of WS contrast in the colon within 4-24 h after administration accurately predicts resolution of ASBO with a sensitivity of 96 per cent and specificity of 98 per cent [57].

In conclusion patients without the above mentioned clinical picture (including all signs of strangulation and/orperitonitis etc.) and a partial SBO or a complete SBO can both undergo non-operative management safely; although, complete obstruction has a higher level of failure [58].

#### Third issue is which conservative management can be adopted and which adjuncts can be used

There are no advantages with the use of long tube decompression compared with the use of nasogastric tubes. (Level of Evidence 1b GoR A)

However early tube decompression, either with long or nasogastric tube, may be beneficial (Level of Evidence 2b GoR C)

The use of Gastrografin in ASBO is safe (in terms of morbidity and mortality) and reduces the need for surgery, the time to resolution of obstruction and the hospital stay (Level of Evidence 1a GoR A)

Gastrografin may be administered on the dosage of 50-150 ml, either orally or via NGT and can be given both at immediately admission or after an attempt of initial traditional conservative treatment of 48 hours (Level of Evidence 1b GoR A)

Oral therapy with magnesium oxide, L. acidophilus and simethicone may hasten the resolution of conservatively treated partial adhesive small bowel obstruction and shorten the hospital stay (Level of Evidence 1b GoR A)

Hyperbaric oxygen (HBO) therapy may be beneficial in non operative management of ASBO, especially in older patients with high anesthesiologic risk (Level of Evidence 2b GoR B)

A prospective RCT comparing tube decompression with either Naso-Gastric Tube or Long intestinal tube, failed to demonstrate any advantage of one type of tube over the other in patients with adhesive SBO [out of 21 patients who ultimately required operation, 13 have been managed with NGT (46%) and 8 with LT (30%) (*p*= 0.16)] [59]. However at operation, 3 patients in the NGT group had ischemic bowel that required resection and, although not proven, the abscence of strangulation in LT group may be attributed to the superior intraluminal decompression provided by LT as compared with NGT. Postoperative complications occurred in 23% of patients treated with NGT versus 38% of patients treated with LT (P = 0.89). Postoperative ileus averaged 6.1 days for NGT patients versus 4.6 days for LT patients (P = 0.44).

Even the 2007 EAST guidelines on SBO management [60] stated that there is no significant difference with regard to the decompression achieved, the success of nonoperative treatment, or the morbidity rate after surgical intervention comparing long tube decompression with the use of nasogastric tubes.

Nevertheless, in conservative treatment for challenging cases of ASBO, the long tube should be placed as soon as possible [61].

Early tube decompression, either with long intestinal tube or just a naso-gastric tube, is therefore advisable in the initial management of non strangulating ASBO, in adjunct with fluid resuscitation and electrolytes imbalances correction.

The first evidence of safety and efficacy of Water-soluble contrast medium (Gastrografin) use in ASBO was from Assalia et al. in the 90s [62]. The first prospective RCT randomised 99 patients with partial ASBO either to 100 ml of Gastrografin administered through the nasogastric tube or conventional treatment. Mean timing of the first stool was 23.3 hours in the control group and 6.2 hours in the patients receiving Gastrografin (p < 0.00001). Ten obstructive episodes (21%) in the control group required operative treatment compared with six (10%) in the trial group (p = 0.12). Mean hospital stay for the patients who responded to conservative treatment was 4.4 days and 2.2 days in the control and trial groups, respectively (p < 0.00001). One patient in each group died after operation. No Gastrografin-related complications were observed.

A further update of this series including 127 patients [63] not only confirmed the same findings in terms of reduction of resolution of the obstruction and of the hospital stay [mean time to first stool 6.2 hours vs 23.5 (p < .0001) and mean hospital stay for unoperated patients 2.7 vs 5.5 days, (p < .0001)], but also showed as well that significantly fewer episodes in the trial group required operation, 10.4% vs 26.7% (p < 0.013).

Further evidence has been showed that the use of hyperosmolar Water-soluble contrast medium (Gastrografin) in ASBO is safe and reduces the need for surgery when conservative treatment fails (after 48 hrs) and in patients showing partial SBO. In the prospective RCT from Choi et al. [64] the patients showing no clinical and radiologic improvement in the initial 48 hours of conservative treatment for non complicated ASBO were randomized to undergo either Gastrografin meal and follow-through study or surgery. Nineteen patients were randomized to undergo Gastrografin meal and follow-through study and 16 patients to surgery.

Gastrografin study revealed partial obstruction in 14 patients. Obstruction resolved subsequently in all of them after a mean of 41 hours. The other five patients underwent laparotomy because the contrast study showed complete obstruction. The use of Gastrografin significantly reduced the need for surgery by 74%. Therefore the use of Gastrografin in ASBO is safe and reduces the need for surgery when conservative treatment fails.

These results have been validated in a further study where 44 episodes of ASBO showing no improvement after 48 hours of conservative management received Gastrografin and out of them 7 underwent becuase of finding of complete obstruction whereas Partial obstruction was demonstrated in 37 other cases, obstruction resolved subsequently in all of them except one patient who required laparotomy because of persistent obstruction [65].

Biondo et al. demonstrated that water-soluble contrast reduces the hospital stay but does not reduce the need for surgery [66]. After randomizing 83 patients with 90 episodes of ASBO to either 100 ml of Gastrografin or control, conservative treatment was successful in 77 episodes (85.6 per cent), among patients treated conservatively hospital stay was shorter in the Gastrografin group (P < 0.001) and all patients in whom contrast medium reached the colon tolerated an early oral diet; however Gastrografin did not reduce the need for operation (P = 1.000).

In another RCT 45 patients with ASBO were randomized to receive either Gastrografin or placebo and 4 patients in each group required surgery but those who received Gastrografin had complete resolution of their ASBO significantly earlier than placebo patients (12 vs 21 h, P = 0.009) and this translated into a median of a 1-day saving in time in hospital (3 vs 4 days, P = 0.03) [67].

A multicenter RCT from Di Saverio et al. [68] was the first which clearly demonstrated a significant reduction of the operative rate in patients with ASBO conservatively managed with adjunct of hyperosmolar Water-soluble contrast medium (Gastrografin), where has been showed a significant reduction of the operative rate and the time to resolution of obstruction, as well as the hospital stay.

Seventy-six patients were randomised to receiving traditional treatment or 150 ml Gastrografin meal via NGT and follow-through study immediately. In the Gastrografin group obstruction resolved subsequently in 31 of 38 cases (81.5%) after a mean time of 6.4 hours. The remaining seven patients were submitted to surgery, and one of them needed bowel resection for strangulation. In the control group, 21 patients were not submitted to surgery (55%), whereas 17 showed persistent untreatable obstruction and required laparotomy: 2 of them underwent bowel resection for strangulation. The difference in operative rate between the groups reached statistical significance (p = 0.013). The time from the hospital admission for obstruction to resolution of symptoms was significantly lower in the Gastrografin group (6.4 vs. 43 hours; p < 0.01). The length of hospital stay revealed a significant reduction in the Gastrografin group (4.7 vs. 7.8 days; p < 0.05). This reduction was more evident in the subset of patients who did not require surgery (3 vs. 5.1 days; p < 0.01).

Again finally regarding the therapeutic value of Gastrografin, the metanalysis from Abbas et al. (6 RCT included) showed that Water-soluble contrast reduces the hospital stay (weighted mean difference --1·84 days; *P <*0·001) [69] but does not reduce the need for surgery [70].

Nevertheless the most recent metanalysis from Branco et al. [71], including overall 7 studies and having added the most recent ones from 2008 and 2009, has proven that WSCA administration is effective in both reducing the need for surgery (OR 0.62; *p *= 0.007) and shortening hospital stay (WMD -1.87 days; *p *< 0.001), without differences in complications and mortality.

Therefore we can confirm that Water soluble contrast (Gastrografin) given in the setting of partial SBO can improve bowel function (time to Bowel Movements), decrease length of stay as well as it reduces the operative rate and is both therapeutic and diagnostic [72].

As further adjuncts needs to be mentioned that oral therapy with magnesium oxide, L. acidophilus and simethicone may hasten the resolution of conservatively treated partial adhesive small bowel obstruction and shorten the hospital stay [73]. In an RCT randomising 128 patients to either control group (intravenous hydration, nasogastric-tube decompression and nothing by mouth) or intervention group (intravenous hydration, nasogastric-tube decompression and oral therapy with magnesium oxide, Lactobacillus acidophilus and simethicone), more patients in the intervention group than in the control group had successful treatment without surgery (91% vs 76%, *p *= 0.03) and the mean hospital stay was significantly longer among patients in the control group than among those in the intervention group (4.2 vs 1.0 days, *p *< 0.001) without differences in complication and recurrence rates.

Hyperbaric Oxygen therapy may be useful in management of adhesive intestinal obstruction associated with abdominal surgery, even in patients who fail to respond to other conservative treatments. HBO therapy may be a preferred option for treatment of patients for whom surgery should be avoided [74].

#### Further matter of debate are how long should NOM be and when it should be discontinued?

Usually NOM, in absence of signs of strangulation or peritonitis, can be prolonged up to 72 hours of adhesive SBO (Level of Evidence 2b GoR C)

After 3 days without resolution, WSCA study or surgery is recommended (Level of Evidence 2b GoR C)

If ileus persists more than 3 days and the drainage volume on day 3 is > 500 ml, surgery for ASBO is recommended (Level of Evidence 2b GoR C)

With closely monitoring and in the absence of signs suggestive of complications, an observation period even longer than 10 days before proceeding to surgical intervention appears to be safe [75].

However at any time, if onset of fever and leukocytosis greater than 15 000/mm3 (predictors of intestinal complications) are observed, then NOM should be discontinued and surgery is recommended.

In the experience from the retrospective series of Cox et al. [76], out of 123 patients initially managed with conservative treatment, 31 of 38 patients requiring surgical intervention for SBO, had so more than 48 h duration after admission and the difference between cases resolving within 48 h and those requiring surgery after 48 h was significant (*p*< 0.001). Therefore most cases of ASBO that will resolve, seem to do so within 48 h of admission.

Fleshner et al. in their RCT comparing conservative management of ASBO with NGT or LT, reported that, between the 21 patients ultimately requiring operation, the mean period between admission and operation was 60 hours in the NGT group versus 65 hours in the LT group [77].

In a series of 35 patients with ASBO, a long intestinal tube was endoscopically placed and the decompression was successful in up to 90% of the cases [78]. Therefore the authors recommend for patients with ASBO, a trial with long tube decompression for 48 to 72 hours. For those who fail a trial with the long tube, laparotomy with enterolysis or bowel resection is indicated.

Contraindications to a trial with long tube decompression include strangulation obstruction, malignant obstruction, incarcerated hernias, foreign body, radiation enteritis, and peritonitis.

In a series of 53 patients with ASBO and treated with long intestinal tube decompression, laparotomy is appropriate after non-response for 7 and 3 days for complete and partial SBO, respectively [79].

From further experiences, if ileus persists more than 3 days and the drainage volume on day 3 is > 500 ml, surgery for ASBO is recommended [80].

The EAST practice management guidelines for SBO recommend that patients without resolution of the SBO by day 3-5 of non-operative management should undergo water soluble study or surgery [81].

#### Finally when deciding between operative or non operative management it would be beneficial to assess the risk of ASBO recurrence after NOM and which factors can predict recurrence of ASBO after NOM

The patients non responders to the long-tube and conservative treatment within 72 hours have a considerable risk of recurrent ASBO (Level of Evidence 2b GoR C)

Risk factors for recurrences are age <40 years and matted adhesion (Level of Evidence 1b GoR A)

Gastrografin use does not affect the recurrences rates or recurrences needing surgery when compared to traditionally conservatively treated patients (Level of Evidence 1b GoR A)

Out of 32,583 patients with an index admission for SBO in 1997 from an US population study [82], 24% had surgery during the index admission and regardless of treatment during the index admission, 81% of surviving patients had no additional SBO readmissions over the subsequent 5 years.

A prospective multicenter study including 286 patients operated on for an adhesive postoperative SBO and followed up for a median time of 41 months. The cumulative incidence of overall recurrence was 15.9%, and for surgically managed recurrence 5.8%. After multivariate analysis, the risk factors for the overall recurrences were age <40 years (hazard ratio HR, 2.97), adhesion or matted adhesion (HR, 3.79) and, for the surgically managed: adhesions or matted adhesions (HR, 3.64), and postoperative surgical complications (HR, 5.63) [83].

Non-operative treatment for adhesions in stable patients results in a shorter hospital stay and similar recurrence and reoperation rates, but a reduced interval to reobstruction when compared with operative treatment [84]. In details patients treated without operation had a 34 per cent readmission rate, compared with 32 per cent for those treated surgically (P not significant), a shorter time to readmission (median 0.7 versus 2.0 years; P < 0.05), no difference in reoperation rate (14 versus 11 per cent; P not significant) and fewer inpatient days over all admissions (4 versus 12 days; P < 0.0001).

In retrospective series of 79 patients with ASBO, out of 23 patients who recovered from ASBO following conservative treatment after 3 days with long intestinal tubes, 16 patients showed recurrent ASBO and half underwent surgery within 3 years [85].

Therefore the patients who did not respond to the long-tube treatment within 72 hours have a significantly higher chance of developing recurrent ASBO.

The same authors in a further study identified 91 patients who recovered from ASBO with nonoperative management after long tube placement and divided them into two groups for follow-up: the recurrence group and the no-recurrence group [86] A significant difference was found in the number of previous ASBO admissions and the duration of long-tube placement (77 hours vs. 43 hours). By multivariate analysis, the duration of long-tube placement was an independent parameter predicting the recurrence of ASBO. Therefore the duration of long-tube placement might serve as a parameter for predicting recurrence of ASBO in patients managed with a long tube.

When addressing the association between type of treatment (surgical versus conservative) and the risk of recurrence, the results of a prospective study with long term follow up showed that the risk of recurrence was significantly lower in patients when the last ASBO episode was surgically treated than when it was nonsurgically treated (RR 0.55) [87]. Subanalyses showed that the relative risk of being reoperated was the same regardless of treatment method for the last episode (RR 0.79). However, the relative risk of being readmitted for ASBO without being operated was significantly lower for patients treated surgically for their last ASBO episode (RR 0.42).

In the series from Williams et al. [88] the frequency of recurrence for those treated nonoperatively was 40.5% compared with 26.8% for patients treated operatively (P < 0.009). Patients treated without operation had a significantly shorter time to recurrence (mean, 153 vs. 411 days; P < 0.004) and had fewer hospital days for their index small bowel obstruction (4.9 vs. 12.0 days; P < 0.0001). However there was no significant difference between early and late recurrent small bowel obstruction in patients treated nonoperatively or operatively, regardless of prior history of abdominal surgery. Logistic regression analysis failed to identify any specific risk factors that were predictors of the success of conservative or surgical management.

The use of Gastrografin does not seem to affect the recurrence rate or speeding up the recurrence after conservatively treated ASBO. In a multicenter RCT, no significant differences in the relapse rate were found when compared to traditional conservative treatment (relapse rate, 34.2% after a mean time to relapse of 6.3 months in the Gastrografin group vs. 42.1% after 7.6 months; *p *= ns) [89].

### - Surgical Treatment: Open and Laparoscopic approach

After 3 days of NOM without resolution of ASBO surgery is recommended (LOE 2c GoR C)

If ileus persists more than 3 days and the drainage volume on day 3 is > 500 ml, surgery for ASBO is recommended (Level of Evidence 2b GoR C)

Also when fever and leukocytosis level (> 15 000/mm3) rises anytime during the course, then surgery is advised GoR C

Open surgery is the preferred method for the surgical treatment of strangulating ASBO and afte failed conservative management (LOE 2c GOR C)

In highly selected group of patients the laparoscopic can be attempted using an open access technique (LOE 2c GOR C)

The access in the left upper quadrant should be safe (LOE 4 GOR C)

Laparoscopic lysis of adhesions should be attempted preferably in case of first episode of SBO and/or anticipated single band adhesion (i.e. SBO after appendectomy or hysterectomy) (LOE 3b GOR C)

A low threshold for open conversion should be maintained if extensive adhesions are found (LOE 2c GOR C)

Conversion to laparoscopic-assisted adhesiolysis (mini-laparotomy with an incision less than 4 cm long) or laparotomy should be considered in those patients presenting with dense or pelvic adhesion (LOE 3b GOR C)

The extent of adhesiolysis is a matter still under debate. The approaches to adhesiolysis for bowel obstruction among general surgeons in the United Kingdom were established in 1993 [90]. Half of all surgeons divided all adhesions to prevent recurrence of bowel obstruction, whereas the other half limited adhesiolysis to only the adhesions responsible for the obstruction.

Adhesions are less after transverse or Pfannenstiel incision in comparison to midline incisions and after surgery for obstetric compared with gynaecological indications [91]. The risk of anterior abdominal wall adhesions increases with the number of previous laparotomies although this relationship is not as evident as the relationship between previous laparotomies and adhesiolysis-induced enterotomy [92,93].

In a prospective study of 1791 patients undergoing benign colorectal surgery (n = 1701) or surgery for small bowel obstruction (n = 90) with 89% having baseline adhesions, the mean time to lyse adhesions was 34 min ranging from 1 to 240 min [94]. Mean time required for lysis of adhesions was about one-fifth of total mean operative time. Notably, 34% of patients had no previous abdominopelvic surgery and presented non-surgical adhesions resulting from intra-abdominal inflammatory and infectious processes associated with benign colorectal diseases including diverticulitis, Crohn's disease and ulcerative colitis.

Higher age and higher number of previous laparotomies appeared to be predictors of the occurrence of inadvertent enterotomy [95]. Patients with three or more previous laparotomies had a 10-fold increase in enterotomy compared with patients with one or two previous laparotomies strongly suggesting more dense adhesion reformation after each reoperation

Historically, laparotomy and open adhesiolysis have been the treatment for patients requiring surgery for small bowel obstruction. Unfortunately, this often leads to further formation of intraabdominal adhesions with approximately 10% to 30% of patients requiring another laparotomy for recurrent bowel obstruction [96].

In animal models laparoscopy has been shown to decrease the incidence, extent, and severity of intraabdominal adhesions when compared with open surgery, thus potentially decreasing the recurrence rate for adhesive small bowel obstruction [97].

Laparoscopy seems to have an advantage above laparotomy in terms of adhesion formation to the abdominal wall and to the operative site [98,99].

Laparoscopic adhesiolysis for small bowel obstruction has a number of potential advantages: (1) less postoperative pain, (2) quicker return of intestinal function, (3) shorter hospital stay, (4) reduced recovery time, allowing an earlier return to full activity, (5) fewer wound complications, and (6) decreased postoperative adhesion formation [100].

However No randomized controlled trial comparing open to laparoscopic adhesiolysis exists up to date, and both the precise indications and specific outcomes of laparoscopic adhesiolysis for adhesive SBO remain poorly understood. The only RCT on laparoscopic adhesiolysis assessed the incidence of chronic abdominal pain after randomization to laparoscopic adhesiolysis or no treatment during diagnostic laparoscopy and it failed to demonstrate any significant differences in terms of pain or discomfort [101].

Although data from retrospective clinical controlled trials suggest that laparoscopy seems feasible and better in terms of hospital stay and mortality reduction, high quality randomised controlled trials assessing all clinically relevant outcomes including overall mortality, morbidity, hospital stay and conversion are lacking [102].

Although the adhesiolysis hospitalization rate has remained constant in USA since 1988, inpatient expenditures have decreased by nearly 10% because of a 15% decrease in the average length of stay (from 11.2 days in 1988 to 9.7 days in 1994) [103]. From this large population Hospital Discharge reports Survey, is derived that laparoscopic less invasive surgical techniques for adhesiolysis, increased over the last years, have contributed to the decreased time required in the hospital for both the surgical procedure itself and the recovery time. However the increased use of laparoscopy during this study period did not appear to be associated with a concomitant reduction in the adhesiolysis hospitalization rate therefore a common denominator may exist between surgical trauma and immune response to foreign bodies.

When deciding between an open or laparoscopic approach, the first consideration is that the surgeon be trained and capable of performing advanced laparoscopy. With regards to patient selection, patients with an acute small bowel obstruction and peritonitis or free air requiring an emergent operation are best managed with a laparotomy. Patients without peritonitis who do not resolve with nonoperative management should be considered for laparoscopic adhesiolysis. In these cases, it is important to consider the bowel diameter, degree of abdominal distention, and location of the obstruction (ie, proximal or distal). Suter et al [104] found that a bowel diameter exceeding 4 cm was associated with an increased rate of conversion: 55% versus 32% (*p *= 0.02). Patients with a distal and complete small bowel obstruction have an increased incidence of intraoperative complications and increased risk of conversion. Patients with persistent abdominal distention after nasogastric intubation are also unlikely to be treated successfully with laparoscopy.

The influence of dense adhesions and the number of previous operations on the success of laparoscopic adhesiolysis is controversial. León et al state that a documented history of severe or extensive dense adhesions is a contraindication to laparoscopy [105]. Navez et al [106] found that patients who had only a previous appendectomy were most likely to be successfully managed with laparoscopy. In contrast, Suter et al found no correlation between the number and or type of previous surgeries and the chance of a successful laparoscopic surgery [107]. Other factors such as an elevated white blood cell count or a fever have not been demonstrated to correlate with an increased conversion rate [Suter et al., Navez et al.]. One group of patients who are good candidates for laparoscopic adhesiolysis are those with a nonresolving, partial small bowel obstruction or a recurrent, chronic small bowel obstruction demonstrated on contrast study [108,109].

In a recent series of 46 patients [110], best results in terms of success rate (91,3%) and no intraoperative bowel perforations, with a relapse free rate of 93,5% after a mean follow up of 46,5 months, can be achieved with the laparoscopic approach when it is used for subgroups of patients with recurrent SBO after abdominal or pelvic surgery, scheduled for elective adhesiolysis, or if the laparoscopic intervention is performed early when the patient had failed to respond to 24 hrs of conservative treatment from the onset of acute SBO.

Perforated or gangrenous bowel is best managed with conversion to either a minilaparotomy or a formal laparotomy. Matted small bowel loops and dense adhesions are also best managed with a formal laparotomy. Navez et al reported that only 10% of obstructions caused by dense adhesions could be treated successfully with laparoscopy. On the other hand, when the cause of obstruction was a single band, laparoscopic adhesiolysis was successful 100% of the time [111].

When other etiologies are found, such as internal hernia, inguinal hernia, neoplasm, inflammatory bowel disease, intussusception, and gallstone ileus, conversion to a minilaparotomy or a formal laparotomy is required.

Inadvertent enterotomy during reopening of the abdomen or subsequent adhesion dissection is a feared complication of surgery after previous laparotomy. The incidence can be as high as 20% in open surgery and between 1% and 100% in laparoscopy [112].

The incidence of intraoperative enterotomies during laparoscopic adhesiolysis ranges from 3% to 17.6%, with most authors reporting an incidence of about 10% [113,114]. Suter et al reported an intraoperative enterotomy incidence of 15.6%, of which 62% were repaired laparoscopically. One of the most dreaded complications of surgery is a missed enterotomy. Although a missed enterotomy can occur after laparotomy, the incidence is higher after laparoscopic surgery. Again Suter et al reported 4 of 47 cases (8.5%) of missed enterotomies requiring reoperation.

The long-term results regarding recurrence are limited, with most series reporting a mean follow-up between 12 and 24 months. Navez et al reported that 85% (29 of 34) of the patients treated laparoscopically were asymptomatic with a mean follow-up of 46 months. The series with the longest follow-up (mean 61.7 months) reported 87.5% (14 of 16) of the patients treated laparoscopically were asymptomatic [115].

Feasibility of diagnostic laparoscopy is ranging from 60% to 100% whilst therapeutic effectiveness of the laparoscopic approach is lower (40-88%). Predictive factors for successful laparoscopic adhesiolysis are: number of previous laparotomies ≤2, non-median previous laparotomy, appendectomy as previous surgical treatment causing adherences, unique band adhesion as phatogenetic mechanism of small bowel obstruction, early laparoscopic management within 24 hours from the onset of symptoms, no signs of peritonitis on physical examination, experience of the surgeon [116].

Surgical operating time is greater in patients who underwent laparoscopic surgery compared to patients who underwent a laparotomy [117,118]. However the duration of laparoscopic procedure is variable ranging from 20 minutes for a simple band adhesion to 2-3 hours for more complex cases [119,120]. Postoperative morbidity is lower in patients who underwent laparoscopic adhesiolysis compared to those who underwent the laparotomic approach. Furthermore a greater rate of morbidity is present in patients who underwent laparotomic conversion; whereas mortality is comparable in the two groups (0-4%). Finally the laparoscopic adhesiolysis can avoid laparotomy, which is itself a cause of new adhesions and bowel obstruction, although some authors noticed a greater incidence of recurrent small bowel obstructions in patients who underwent laparoscopy compared to those in which a laparotomy was performed [121-124].

In a large review of 308 patients from 35 centres [125] over 8 years the 'successful' laparoscopy rate was 54.6% and the conversion to laparotomy rate was 45.4%. There were significantly more successes among patients with a history of one or two laparotomies than among those with three or more (56% vs 37%; *p *< 0.05). Furthermore the rate of success was significantly higher (*p *< 0.001) in patients operated on early (<24 h) and in patients with bands (54%), than in those with matted adhesions (31%).

In a French experience the laparoscopic approach, with a conversion rate of 31%, did not show any influence on the early postoperative mortality (*P *= .7) nor on morbidity (*P *= .4) [126].

Although a laparoscopic approach has been proposed to decrease the incisional trauma and to lower the rate of recurrence, a slightly higher but nonstatistically significant rate of recurrences in the laparoscopic approach has been observed. Probably, further several different even smaller incisions and a mandatory identical parietal and visceral adhesiolysis as laparotomy do not decrease the magnitude of the peritoneal trauma [127].

The largest and most significant large population review from US identified from the 2002 National Inpatient Sample 6,165 patients with intestinal obstruction undergoing open (OLA) and laparoscopic lysis of adhesions (LLA) [128]. 88.6% underwent OLA and 11.4% had LLA. Conversion was required in 17.2% of LLA patients. Unadjusted mortality was equal between LLA and conversion (1.7%) and half the rate compared with OLA (3.4%) (p = 0.014). The odds of complications in the LLA group (intention to treat) were 25% less than in the OLA (p = 0.008). The LLA group had a 27% shorter LOS (p = 0.0001) and was 9% less expensive than the OLA group (p = 0.0003). There was no statistical significant difference for LOS, complications, and costs between the conversion and OLA groups.

The comparably low conversion rate of 17% by Mancini et al. in this study may be explained by the low initial percentage (11%) of patients treated laparoscopically, indicating a positive selection of patients amenable to successful laparoscopic adhesiolysis.

Szomstein and colleagues [129] summarized data on conversion rates for laparoscopic lysis of adhesions and reported a range from 6.7% to 41%. The benefits and advantages of laparoscopic approach for lysis of adhesions are highlighted in this review of 11 series including 813 patients. They have found that 63% of the length of a laparotomy incision is involved in adhesion formation to the abdominal wall. Furthermore, the incidence of ventral hernia after a laparotomy ranges between 11% and 20% versus the 0.02%-2.4% incidence of port site herniation. Additional benefits of the minimally invasive approaches include a decreased incidence of wound infection and postoperative pneumonia and a more rapid return of bowel function resulting in a shorter hospital stay. In long-term follow up, the success rate of laparoscopic lysis of adhesions remains between 46% and 87%. Operative times for laparoscopy range from 58 to 108 minutes; conversion rates range from 6.7% to 43%; and the incidence of intraoperative enterotomy ranges from 3% to 17.6%. The length of hospitalization is 4-6 days in most series. In this review again contraindications to the minimally invasive technique include the following: (1) massive abdominal distension that precludes entry into the peritoneal space and limits adequate working space; (2) the presence of peritonitis with the need for bowel resection and bowel handling in a highly inflamed environment; (3) hemodynamic instability; (4) severe comorbid conditions such as heart and lung diseases that preclude the use of pneumoperitoneum; and (5) finally, but certainly not the least important, the surgeon's comfort level.

An interesting although small review of 93 patients with ASBO from a community teaching hospital [130], divided into successful laparoscopy (66 patients [71%]), secondary conversion (24 [26%]), and primary laparotomy (three patients), showed that patients with successful laparoscopy had more simple adhesions (57%), fewer prior operations, and lower ASA class. Operative time was shortest in the laparoscopy group (74.3 ± 4.4 min), as was the duration of both intensive care unit and hospital stay. Mortality was 6%, regardless of operative technique. The author's conclusion confirmed that the parameters associated with successful laparoscopic management of SBO are the presence of isolated bands, lower ASA scorse, younger age, fewer prior operations, and a shorter duration of SBO obstruction before the operation. Reasons for primary laparotomy included a state of prolonged ileus with progressive abdominal distension and a higher number or more extensive previous operations. Reasons for converting to open adhesiolysis following initial laparoscopy were inadequate laparoscopic control due to intestinal distension, extensive adhesions, iatrogenic intestinal perforation and the presence of necrotic segments of the small bowel upon initial laparoscopy, requiring secondary open resection.

Zerey et al. [131] reported a series of 33 patients underwent laparoscopic adhesiolysis secondary to a SBO. Twenty-nine patients (88%) were successfully treated laparoscopically. Mean procedural time was 101 minutes (range, 19-198 minutes). Only one patient had a recurrent SBO 8 months postoperatively managed by repeat laparoscopic lysis of adhesions. Mean postoperative stay was 6 days.

In another report of 65 patients submitted to laparoscopic adhesiolysis (40 for acute obstruction and 25 for chronic or recurrent transit disturbances) the procedure was completed by laparoscopy in 52 patients (conversion rate: 20%) and after a mean follow up of 48 months has been observed a 15.4% rate of symptomatic recurrences, while surgical recurrences have been 4.6% [132].

In a series of 17 patients scheduled for elective adhesiolysis [133], laparoscopic treatment was successful in 14 patients (82.4%) and two recurrences of small bowel obstructions were noted over a mean follow-up period of 61.7 months. In a similar series of elective laparoscopic treatment of 25 patients with recurrent small bowel obstruction, complete laparoscopic adhesiolysis was feasible in 18 patients (72%) and no recurrence of small bowel obstruction over a mean follow-up period of 41 months have been observed [134]. In this series conversion to laparoscopic-assisted adhesiolysis (mini-laparotomy with an incision less than 4 cm long) was required in 6 patients (24%) because of dense adhesion or the technical difficulties due to adhesion in the pelvic cavity.

Leon et al. reported a 35% conversion rate in a series of 40 patients and at median follow-up of 12 months, 21 of 26 patients managed laparoscopically or with laparoscopic-assisted procedures remained asymptomatic [135].

A review in 2007 show that laparoscopic management of SBO is successful in 66% of patients with a conversion rate of 33.5% [136].

Operative technique has capital role for a successful laparoscopic treatment [137]. The initial trocar should be placed away (alternative site technique) from the scars in an attempt to avoid adhesions. Some investigators have recommended the use of computed tomography scan or ultrasonography to help determine a safe site for the initial trocar insertion.

The left upper quadrant is often a safe place to gain access to the abdominal cavity. Alternatively a 10 mm port can be inserted in the left flank with two additional 5 mm ports in the left upper and lower quadrant. Therefore, by triangulating 3 ports aimed at the right lower quadrant, a good exposure and access to the right iliac fossa can be obtained and a technique running the small bowel in a retrograde fashion, starting from the ileocecal valve (decompressed intestine) proximally towards the transition point between collapsed and dilated loops.

The open (Hasson) approach under direct vision is the more prudent. Once safe access is obtained, the next goal is to provide adequate visualization in order to insert the remaining trocars. This often requires some degree of adhesiolysis along the anterior abdominal wall. Numerous techniques are available, including finger dissection through the initial trocar site and using the camera to bluntly dissect the adhesions. Sometimes, gentle retraction on the adhesions will separate the tissue planes. Most often sharp adhesiolysis is required. The use of cautery and ultrasound dissection should be limited in order to avoid thermal tissue damage and bowel injury.

Strickland have reported an incidence of 10% enterotomies during exploration and adhesiolysis in 40 patients treated laparoscopically for acute SBO. However an even higher proportion of the patients had enterotomies after conversion (23%) [138]. Furthermore formal laparotomy was avoided in 68% of these patients and earlier return of bowel function and a shorter postoperative length of stay, with lower overall costs was achieved with laparoscopic treatment. The risk of enterotomy can be reduced if meticulous care is taken in the use of atraumatic graspers only and if the manipulation of friable, distended bowel is minimized by handling the mesentery of the bowel whenever possible. In fact to handle dilated and edematous bowel during adhesiolysis is dangerous and the risk increases with a long lasting obstruction; therefore early operation is advisable as one multicenter study showed that the success rate for early laparoscopic intervention for acute SBO was significantly higher after a shorter duration of symptoms (24 h vs 48 h) [139].

Maintaining a low threshold for conversion to laparotomy in the face of extensive adhesions will further decrease the risk of bowel injury.

After trocar placement, the initial goal is to expose the collapsed distal bowel. This is facilitated with the use of angled telescopes and maximal tilting/rotating of the surgical table. It may also be necessary to move the laparoscope to different trocars to improve visualization. If necessary, the small bowel mesentery (instead of the bowel wall) should be grasped in order to manipulate the bowel. Sharp dissection with the laparoscopic scissors should be used to cut the adhesions. Only pathologic adhesions should be lysed. Additional adhesiolysis only adds to the operative time and to the risks of surgery without benefit. The area lysed should be thoroughly inspected for possible bleeding and bowel injury.

In conclusion, careful selection criteria for laparoscopy [140] may be: (1) proximal obstruction, (2) partial obstruction, (3) anticipated single band, (4) localized distension on radiography, (5) no sepsis, (6) mild abdominal distension and last but not least (7) the experience and laparoscopic skills of the surgeon.

The experts panel also agreed, as from the cited studies, that laparoscopic lysis of adhesions should be attempted preferably in case of first episode of SBO and/or anticipated single band adhesion (i.e. SBO after appendectomy or hysterectomy).

Furthermore the experts highlighted that an open port access should be attempted, and gaining the access in the left upper quadrant should be safe. However a large consensus has been reached in recommending a low threshold for open conversion if extensive adhesions are found.

### - Prevention

We do need to prevent ASBO (LOE 2b GoR B)

Hyaluronic acid-carboxycellulose membrane and icodextrin are able to reduce adhesions (respectively LOE 1a GOR A and LOE 1b GOR A).

Icodextrin may reduce the risk of re-obstruction for ASBO (LOE 1 b GOR A).

Hyaluronic acid-carboxycellulose can not reduce the need of surgery for ASBO (LOE 1a GOR A).

A systematic review including a total of 446,331 abdominal operations found an overall incidence of SBO of 4.6% [141]. The risk of SBO was highly influenced by the type of procedure, with ileal pouch-anal anastomosis being associated with the highest incidence of SBO (19.3%), followed by open colectomy (9.5%). Gynecological procedures were associated with an overall incidence of 11.1% and ranged from 23.9% in open adnexal surgery to 0.1% after cesarean section.

Adhesions and ASBO are extremely common and the cumulative recurrence rate for patients operated once for ASBO is 18% at 10 years and 29% at 30 years as shwon in a long term follow up cohort study. Cumulative recurrence rate reaches 81% for patients with 4 or more admissions [142].

Another multicer prospective study [143] showed that the cumulative incidence of overall recurrence of ASBO was 15.9% after a median follow up of 41 months and for surgically managed recurrences it was 5.8%. Therefore, in view of the incidence of adhesions and recurrence rates of ASBO as well as of the magnitude of the medical problems and financial burden related to adhesions, prevention or reduction of postoperative adhesions in an important priority. Even though awareness of this problem is widely agreed among surgeons and gynaecologists, uncertainty still exists about the treatment and prophylactic strategies for dealing with adhesions [144]. A recent national survey among Dutch surgeons and surgical trainees [145] showed that underestimation of the extent and impact of adhesions resulted in low knowledge scores and Lower scores correlated with more uncertainty about indications for antiadhesive agents which, in turn, correlated with never having used any of these agents. Several articles on adhesion barriers have been published but several controversies such as the effectiveness of available agents and their indication in general surgical patients still exist. Most of the available literature is based on gynecologic patients. For general surgical patients no recommendations or guidelines exist.

Any prevention strategy should be safe, effective, practical, and cost effective. A combination of prevention strategies might be more effective [146].

The prevention strategies can be grouped into 4 categories: general principles, surgical techniques, mechanical barriers, and chemical agents.

#### General principles

Intraoperative techniques such as avoiding unnecessary peritoneal dissection, avoiding spillage of intestinal contents or gallstones [147], and the use of starch-free gloves [148,149] are basic principles that should be applied to all patients. In a large systematic review [150], the closure of the peritoneum, spillage and retention of gallstones during cholecystectomy, and the use of starched gloves all seems to increase the risk for adhesion formation.

#### Surgical techniques

The surgical approach (open vs laparoscopic surgery) plays an important role in the development of adhesive SBO.

In the long term follow up study from Fevang et al. [151] the surgical treatment itself decreased the risk of future admissions for ASBO, even though the risk of new surgically treated ASBO episodes was the same regardless of the method of treatment (surgical vs conservative).

The technique of the procedure (open vs. laparoscopic) also seems to play a major role in the development of adhesive SBO. The incidence was 7.1% in open cholecystectomies vs. 0.2% in laparoscopic; 15.6% in open total abdominal hysterectomies vs. 0.0% in laparoscopic; and 23.9% in open adnexal operations vs. 0.0% in laparoscopic. There was no difference in SBO following laparoscopic or open appendectomies (1.4% vs. 1.3%) [152].

In most abdominal procedures the laparoscopic approach is associated with a significantly lower incidence of adhesive SBO or adhesion-related re-admission. In a collective review of the literature the incidence of adhesion-related re-admissions was 7.1% in open versus 0.2% in laparoscopic cholecystectomies, 9.5% in open versus 4.3% in laparoscopic colectomy, 15.6% in open versus 0% in laparoscopic total abdominal hysterectomy, and 23.9% in open versus 0% in laparoscopic adnexal surgery. Only in appendectomies there was no difference between the two techniques [153].

There is some class I evidence in obstetrics supporting the theory that suturing the peritoneum increases the risk of adhesions [154]. It is therefore prudent to avoid peritoneal closure during laparotomies.

#### Mechanical barriers

In theory, inert materials that prevent contact between the damaged serosal surfaces for the first few critical days allow separate healing of the injured surfaces and may help in the prevention of adhesion formation. Various bioabsorbable films or gels, solid membranes, or fluid barrier agents have been tested experimentally and in clinical trials.

Hyaluronic acid/carboxymethylcellulose (Seprafilm) is the most extensively tested adhesion prevention agent in general surgery. Its safety with regard to systemic or specific complications has been established in many studies, including a safety study of 1,791 patients with abdominal or pelvic surgery, however there are concerns about a higher incidence of anastomotic leaks in cases in which the film is placed directly around the anastomosis [155].

Several prospective randomized controlled trials showed efficacy in reducing the incidence and extent of postoperative adhesions. In a prospective, randomized, multicenter, double-blind study of 175 evaluable patients with colectomy and ileoanal pouch procedure, compared Seprafilm with controls, The Seprafilm group had significantly fewer and less severe adhesions and well as of reduced extent [156].

A further prospective multicenter study, randomized 71 patients undergoing Hartmann's resection into a Seprafilm and a control group: although the incidence of adhesions did not differ significantly between the study groups, the Seprafilm group showed a significant reduction of the severity of adhesions [157].

Cohen et al, in a prospective multicenter trial, randomized 120 patients with colectomy and ileal pouch surgeries into a Seprafilm and a control group [158]. The outcomes included incidence and severity of adhesions and were assessed laparoscopically by a blinded observer at a second surgery 8 to 12 weeks later for ileostomy closure. Treatment with Seprafilm significantly reduced the incidence and severity of adhesions.

Kumar et al in a recent Cochrane collective review of 6 randomized trials with nongynecologic surgical patients found that Seprafilm significantly reduced the incidence of adhesions (OR, .15; 95% CI, .05-.43; *P *< .001) and the extent of adhesion (mean difference, --25.9%; 95% CI, --40.56 to --11.26; *P *< .001) [159].

Although there is satisfactory class I evidence that Seprafilm significantly reduces the incidence and severity of postoperative adhesions, there is fairly limited work on the effect of this adhesion reduction on the incidence of SBO.

Fazio et al in a prospective, randomized, multicenter, single-blind study of 1,791 patients with intestinal resection compared Seprafilm with no treatment intervention. There was no difference between the Seprafilm and control group in the overall incidence of SBO (12% vs 12%). However, the incidence of SBO requiring surgical intervention was significantly lower in the Seprafilm group (1.8% vs 3.4%; *P *< .05). This was an absolute reduction of 1.6% and a relative reduction of 47%. Stepwise multivariate analysis showed that the use of Seprafilm was the only independent factor for reducing SBO requiring reoperation [160].

Kudo et al in a nonrandomized study of 51 patients who underwent transabdominal aortic aneurysm surgery, analyzed the incidence of early SBO in patients who had Seprafilm applied and in control patients with no treatment. The incidence of early SBO was 0% in the Seprafilm group and 20% in the control group (*P *< .05) [161].

A dutch RCT including 71 patients requiring a Hartmann procedure for sigmoid diverticulitis or obstructed rectosigmoid were randomized to either intraperitoneal placement of the antiadhesions membrane under the midline during laparotomy and in the pelvis, or as a control [162].

The incidence of adhesions did not differ significantly between the two groups, but the severity of adhesions was significantly reduced in the Seprafilm group both for the midline incision and for the pelvic area. Complications occurred in similar numbers in both groups.

A recent systematic Review and Meta-analysis [163] including 4203 patients showed that incidence of grade 0 adhesions among Seprafilm-treated patients was statistically significantly more than that observed among control group patients. There was no significant difference in the incidence of grade 1 adhesions between Seprafilm and control groups. The severity of grade 2 and grade 3 adhesions among Seprafilm-treated patients was significantly less than that observed among control group patients. The incidence of intestinal obstruction after abdominal surgery was not different between Seprafilm and control groups. Using Seprafilm significantly increased the incidence of abdominal abscesses and anastomotic leaks.

In a Cochrane review of 7 RCT, six compared hyaluronic acid/carboxymethyl membrane (HA/CMC) and one 0.5% ferric hyaluronate gel against controls. HA/CMC reduced the incidence of adhesions with reduced extent and severity [164]. However there was no reduction of intestinal obstruction needing surgical intervention with comparable overall morbidity and mortality. The study of 0.5% ferric hyaluronate gel was prematurely terminated and no valid conclusions could be made but there was a higher incidence of overall morbidity and ileus. Therefore authors' conclusions were that the use of HA/CMC membrane reduces incidence, extent and severity of adhesions which may, theoretically, have implications in re-operative abdominal surgery. There is no evidence that the incidence of intestinal obstruction or need for operative intervention is reduced. HA/CMC appears to be safe but there may be a risk of leak when wrapped around an anastomoses.

Oxidized regenerated cellulose (Interceed) is a mechanical barrier that forms a gelatinous protective coat and breaks down and is absorbed within 2 weeks. This product has been studied in numerous prospective randomized studies in open or laparoscopic gynecologic surgeries. It has been shown to be safe and effective in reducing adhesions. The first study was a prospective, randomized, multicenter, clinical trial that evaluated the efficacy of Interceed in reducing adhesions in humans [165]. Infertility patients (n = 74) with bilateral pelvic sidewall adhesions were studied at treatment laparotomy and "second-look" laparoscopy to determine Interceed's effectiveness. It did show a significant reduction of incidence, extent, and severity of postsurgical pelvic adhesions.

In the second prospective, randomized, controlled clinical study, 21 women underwent a second-look laparoscopy 2-11 weeks after standardized laparoscopic electrosurgical treatment for polycystic ovarian syndrome [166]. Following bilateral ovarian treatment, one ovary was randomly chosen to have Interceed applied to its surface using a specially designed applicator, with the other ovary serving as a control. Peri-adnexal adhesions of significant extent and severity developed in 57% of the women and 38% of the adnexa. The incidence of adhesions on the Interceed-treated side was 43%, while on the control side it was 33%. In addition, the extent and severity of the adhesions appeared to be similar on the Interceed-treated and control side.

In a prospective randomized study of 134 women undergoing adhesiolysis by laparotomy, and having applied Interceed on one sidewall and left the opposite side uncovered, the incidence and severity of adhesions were evaluated at a second-look laparoscopy 10 days to 14 weeks after surgery and Interceed significantly reduced the incidence and extent of adhesions [167]. The Nordic Adhesion Prevention Study group in a multicenter, prospective, randomized, blinded study of 66 women undergoing adhesiolysis of 132 ovaries used Interceed around the adnexa on one side and left the other side uncovered. The incidence and severity of adhesions were assessed at a second-look laparoscopy 4 to 10 weeks after the initial surgery and the results showed that Interceed significantly reduced the incidence, extent, and severity of adhesions [168]. A meta-analysis of 7 randomized studies showed that Interceed decreased the incidence of adhesions by 24.2% ± 3.3% (*P *< .001) when compared with untreated sites [169]. A more recent meta-analysis also concluded that Interceed reduced the incidence and severity of adhesions after open or laparoscopic gynecologic surgery [170].

Expanded polytetrafluoroethylene (Gore-Tex, Preclude; W.L. Gore & Associates, Hertogenbosch, The Netherlands): It is an inert, nonabsorbable permanent membrane that needs to be removed a few days after application. It has been studied mainly in gynecologic surgeries with favorable results [171]. Its usefulness is limited because of the need to be removed surgically at a later stage.

Various Bioabsorbable gels have been developed and tested, but most have been abandoned or withdrawn because of safety issues or a lack of efficacy. SprayGel is a sprayable hydrogel that adheres to the tissues for a period of 5 to 7 days. After several days it is hydrolyzed into water-soluble molecules and is absorbed. Safety of SprayGel has been shown in a few gynecologic and colorectal studies, however although early preliminary clinical trials showed its effectiveness, a larger-scale study was stopped owing to a lack of efficacy [172].

Finally a systematic review of barrier agents for adhesion prevention after gynaecological surgery assessed the effect of physical barriers used during pelvic surgery in women of reproductive age on pregnancy rates, pelvic pain, or postoperative adhesion reformation [173]. The authors' conclusions were that the absorbable adhesion barrier Interceed reduces the incidence of adhesion formation following laparoscopy and laparotomy. Gore-Tex may be superior to Interceed in preventing adhesion formation but its usefulness is limited by the need for suturing and later removal. There was no evidence of effectiveness of Seprafilm and Fibrin sheet in preventing adhesion formation.

#### Chemical/Fluid agents

Fluid agents have the theoretical advantage of covering more potential sites of adhesion formation than mechanical barriers.

A systematic review updated at 2006 [174], regarding fluids and pharmacological agents for adhesion prevention after gynaecological surgery, found insufficient evidence for the use of the following agents: steroids, icodextrin 4%, SprayGel and dextran in improving adhesions following surgery. There was some evidence that hyaluronic acid agents may decrease the proportion of adhesions and prevent the deterioration of pre existing adhesions but the need of further studies was advocated.

The most widely studied and the only Food and Drug Administration-approved adhesion-prevention fluid agent in laparoscopic surgery is Adept (Baxter Healthcare, Deerfield, IL). Adept (icodextrin 4% solution) is used as an irrigant fluid throughout surgery and at the end of surgery 1,000 mL is instilled and left in the peritoneal cavity. The fluid remains in the peritoneal cavity for several days and separates the damaged surfaces during the critical period of adhesion formation. A large multicenter, prospective, randomized, double-blind study by Brown et al [175] compared Adept (N = 203) with lactated Ringer's solution (N = 199), in women undergoing laparoscopic gynecologic surgery for adhesiolysis. The study patients returned for a second laparoscopy within 4 to 8 weeks. Adept was significantly more likely to reduce adhesions and improve fertility scores than lactated Ringer's solution.

A multicenter RCT compare intraperitoneal 4% icodextrin (ID) solution with lactated Ringer's solution (LRS) on adhesion formation after Hartmann's procedure [176]. The adhesiolysis surgery time during Hartman's reversal was used as a marker of the severity of adhesions. On completion of 17 eligible patients, an interim analysis was performed. There were no complications following the use of 4% ID solution. The mean (SD) total adhesiolysis times in patients treated with 4% ID solution and LRS were 30.8 (18.0) min and 47.6 (45.7) min, respectively. The mean reduction of 16.8 min, although greater than expected, was not statistically significant (P = 0.33) because of the large variance in adhesiolysis times. However in interpreting the results of this study, has to be highlighted that it was underpowered to meet the study end-point.

The most recent Italian RCT [177] on use of icodextrin 4% solution for prevention of postoperative abdominal adhesions after laparotomic operation for small bowel obstruction caused by adherences, included 169 patients randomised to either Icodextrin 4% or control and demonstrated a significant (p < 0.05) reduction of ASBO recurrences in the study group after a mean follow up period of 42 months, as well as a trend, although not statistically significant, in decreasing the incidence of recurrences needing surgery and the severity of adhesions.

The ARIEL registry [178] (multicentre Adept Registry for Clinical Evaluation) was established to gather clinical experiences in the use of icodextrin 4% solution, an approved adhesion-reduction agent, during routine general surgery. General surgeons from five European countries completed anonymised data collection forms for patients undergoing laparotomy or laparoscopy. Surgeons recorded patient demographics, use of icodextrin 4% solution and adverse events, and made subjective assessments of ease of use and patient acceptability with the agent. This registry showed that the volumes of icodextrin 4% solution used as an irrigant and instillate were in line with recommendations (1-l instillation and 100 ml every 30 min for irrigation). Surgeons considered the agent to be easy to use and acceptable to patients. The reported frequencies of adverse events were in line with those published in the literature for surgical procedures, supporting the good safety profile of this agent.

Intergel solution (Lifecore Biomedical, Inc, Chaska, MN), which contains .5% ferric hyaluronate, is another solution used for adhesion prevention. In preliminary studies it has been shown to reduce the number, severity, and extent of adhesions in peritoneal surgery [179]. However, the use of Intergel in abdominal surgery in which the gastrointestinal tract was opened led to an unacceptably high rate of postoperative complications [180].

#### Miscellanous

An interesting experimental finding is the reduction of both number and type of adhesions after postoperative stimulation of gastrointestinal motility by a prokinetic agent [181].

Finally merits mention that peritoneal infusion with cold saline has shown to decrease the degree of postoperative intra-abdominal adhesion formation in an animal model [182].

## Audience and Panelists Remarks

PREVENTION:

"the cited metanalysis contains only one RCT. So change LOE from 1a to 1b"

VAN GOOR

"the statement PATIENTS WHO HAD SURGERY WITHIN 6 WEEKS, should be taken out from the exclusion criteria for NOM"

PINNA AD, SUGABAKER

"the CT scan findings and the factors predictive of surgery, derived from the paper WJS 2010 from the group of Mayo Clinic - M. Sarr, should be defined further clarifying their OR, from the more weak (lack of feaces sign) to the strongest. Should also be highlighted that the combination of the 4 factors has an higher OR (16...) and therefore the combined presence has an higher GoR"

M. VALENTINO

"the weak evidence of the value of the small bowel faeces sign should be highlighted"

M. VALENTINO

"the citation of the paper studying the effect of high oxygen on the conservative management of ASBO should be included in the paper and this effect of high oxygen should included in the guidelines"

http://www.ncbi.nlm.nih.gov/pubmed/18613394

VAN GOOR

"change the definition if ILEUS persist with the definition if ASBO persist, since ileus in english refers usually to postoperative ileus"

P. SUGARBAKER

"I would be more conservative with patients with recurrent ASBO. The limit of 72 hours for the indications for surgery should be delayed for the patients with recurrent ASBO"

C. BENDINELLI AND PINNA AD

## Conclusions

ASBO is a common disease. Non operative management should be attempted in absence of signs of peritonitis or strangulation. WSCM is safe and has a definite role in diagnosis (for predicting the resolution or need for surgery) and therapy (for reducing the operative rate and shortening time to resolution of symptoms and hospital stay). Open surgery remains the safest and most effective operative approach. Prevention with hyaluronic acid-carboxycellulose membrane or icodextrin, has actually a capital relevance.

## Competing interests

The authors declare that they have no competing interests.

## Authors' contributions

**FC, SDS**: conception and design of the study; organised the consensus conference; preparation of the draft; merged the committee preliminary statements with the observations and recommendations from the panel,

summarised the discussion on standards of diagnosis and treatment for ASBO

**SDS, FC **manuscript writing, drafting and review.

**FC, SDS, MDK, JJ **organised the consensus conference, merged the committee preliminary statements with the observations and recommendations from the panel, critically contributed to the consensus statements

**MDK, WLB, LA, VM, HVG, EEM, JJ **contributed to critical discussion of the draft

**All authors **read and approved the final manuscript

## References

[B1] Parker C Ellis H Moran BJ Postoperative adhesions: ten-year follow-up of 12,584 patients undergoing lower abdominal surgery Dis Colon Rectum 2001 44 822 830 10.1007/BF02234701 11391142

[B2] Ellis The magnitude of adhesion related problems Ann Chir Gynaecol 1998 87 9 11 9598223

[B3] Hershlag A Diamond MP DeCherney AH Adhesiolysis Clin Obstet Gynecol 1991 34 395 401 10.1097/00003081-199106000-00023 1831077

[B4] Monk BJ Berman ML Montz FJ Adhesions after extensive gynecologic surgery: clinical significance, etiology, and prevention Am J Obstet Gynecol 1994 170 1396 1403 8178880 10.1016/s0002-9378(94)70170-9

[B5] Milingos S Kallipolitis G Loutradis D Adhesions: laparoscopic surgery versus laparotomy Ann N Y Acad Sci 2000 900 272 285 10.1111/j.1749-6632.2000.tb06239.x 10818415

[B6] Vrijland WW Jeekel J van Geldorp HJ Abdominal adhesions: intestinal obstruction, pain, and infertility Surg Endosc 2003 17 1017 1022 10.1007/s00464-002-9208-9 12632122

[B7] Ray NF Denton WG Thamer M Henderson SC Perry S Abdominal adhesiolysis: inpatient care and expenditures in the United States in 1994 J Am Coll Surg 1998 186 1 9 10.1016/S1072-7515(97)00127-0 9449594

[B8] Foster NM McGory ML Zingmond DS Ko CY Small bowel obstruction: a population-based appraisal J Am Coll Surg 2006 203 170 176 10.1016/j.jamcollsurg.2006.04.020 16864029

[B9] Menzies D Peritoneal adhesions. Incidence, cause, and prevention Surg Annu 1992 24 Pt 1 27 45 1727325

[B10] Luijendijk RW de Lange DC Wauters CC Hop WC Duron JJ Pailler JL Camprodon BR Holmdahl L van Geldorp HJ Jeekel J Foreign material in postoperative adhesions Ann Surg 1996 223 3 242 8 10.1097/00000658-199603000-00003 8604903 PMC1235111

[B11] Coleman G McLain AD Moran BJ Impact of previous surgery on time taken for incision and division of adhesions during laparotomy Dis Colon Rectum 2000 43 1297 1299 10.1007/BF02237441 11005501

[B12] Van Der Krabben A Dijkstra FR Nieuwenhuijzen M Morbidity and mortality of inadvertent enterotomy during adhesiotomy Br J Surg 2000 87 467 471 10.1046/j.1365-2168.2000.01394.x 10759744

[B13] EAST Practice Parameter Workgroup for Management of Small Bowel Obstruction Practice management guidelines for small bowel obstruction 2007 Chicago (IL): Eastern Association for the Surgery of Trauma (EAST) 42

[B14] Parker MC Ellis H Moran BJ Postoperative adhesions: ten-year follow-up of 12,584 patients undergoing lower abdominal surgery Dis Colon Rectum 2001 44 822 830 10.1007/BF02234701 11391142

[B15] Parker C Wilson MS Menzies D The SCAR-3 study: 5-year adhesion-related readmission risk following lower abdominal surgical procedures Colorectal Dis 2005 7 551 558 10.1111/j.1463-1318.2005.00857.x 16232234

[B16] Luijendijk RW de Lange DC Wauters CC Foreign material in postoperative adhesions Ann Surg 1996 223 242 248 10.1097/00000658-199603000-00003 8604903 PMC1235111

[B17] Tortella BJ Lavery RF Chandrakantan A Incidence and risk factors for early small bowel obstruction after celiotomy for penetrating abdominal trauma Am Surg 1995 61 956 958 7486425

[B18] Stewart RM Page CP Brender J The incidence and risk of early postoperative small bowel obstruction: A cohort study Am J Surg 1987 154 643 647 10.1016/0002-9610(87)90234-0 3425811

[B19] Barkan Howard Webster Steven Steven Ozeran Factors predicting the recurrence of adhesive small-bowel obstruction The American Journal of Surgery October 1995 170 4 361 365 10.1016/s0002-9610(99)80304-3 7573729

[B20] Barkan Webster S Ozeran S Factors predicting the recurrence of adhesive small-bowel obstruction Am J Surg 1995 170 361 365 10.1016/S0002-9610(99)80304-3 7573729

[B21] Duron JJ Silva NJ du Montcel ST Adhesive postoperative small bowel obstruction: incidence and risk factors of recurrence after surgical treatment: a multicenter prospective study Ann Surg 2006 244 750 757 10.1097/01.sla.0000225097.60142.68 17060768 PMC1856591

[B22] Williams SB Greenspon J Young HA Orkin BA Small bowel obstruction: conservative vs. surgical management Dis Colon Rectum 2005 48 6 1140 6 10.1007/s10350-004-0882-7 15906139

[B23] Duron JJ du Montcel ST Berger A Muscari F Hennet H Veyrieres M Hay JM French Federation for Surgical Research. Prevalence and risk factors of mortality and morbidity after operation for adhesive postoperative small bowel obstruction Am J Surg 2008 195 6 726 34 10.1016/j.amjsurg.2007.04.019 18367136

[B24] Duron JJ Silva NJ du Montcel ST Berger A Muscari F Hennet H Veyrieres M Hay JM Adhesive postoperative small bowel obstruction: incidence and risk factors of recurrence after surgical treatment: a multicenter prospective study Ann Surg 2006 244 5 750 7 10.1097/01.sla.0000225097.60142.68 17060768 PMC1856591

[B25] Miller G Boman J Shrier I Gordon PH Natural history of patients with adhesive small bowel obstruction Br J Surg 2000 87 9 1240 7 10.1046/j.1365-2168.2000.01530.x 10971435

[B26] Fevang BT Fevang J Lie SA Søreide O Svanes K Viste A Long-term prognosis after operation for adhesive small bowel obstruction Ann Surg 2004 240 2 193 201 10.1097/01.sla.0000132988.50122.de 15273540 PMC1356393

[B27] Di Saverio S Tugnoli G Orlandi PE Catena F A 73-year-old man with long-term immobility presenting with abdominal pain PLoS Med 2009 6 e1000092 10.1371/journal.pmed.1000092 19597540 PMC2702819

[B28] Thompson William M Accuracy of Abdominal Radiography in Acute Small-Bowel Obstruction: Does Reviewer Experience Matter? AJR 2007 188 W233 W238 10.2214/AJR.06.0817 17312028

[B29] Schmutz GR Benko A Fournier L Peron JM Morel E Chiche L Small bowel obstruction: role and contribution of sonography Eur Radiol 1997 7 1054 1058 10.1007/s003300050251 9265673

[B30] Grassi R Romano S D'Amario F The relevance of free fluid between intestinal loops detected by sonography in the clinical assessment of small bowel obstruction in adults Eur J Radiol 2004 50 1 5 14 10.1016/j.ejrad.2003.11.009 15093230

[B31] Obuz F Terzi C Sokmen S Yilmaz E Yildiz D Fuzun M The efficacy of helical CT in the diagnosis of small bowel obstruction Eur J Radiol 2003 48 3 299 304 10.1016/S0720-048X(02)00382-0 14652150

[B32] Trésallet C Lebreton N Royer B Leyre P Godiris-Petit G Menegaux F Improving the management of acute adhesive small bowel obstruction with CT-scan and water-soluble contrast medium: a prospective study Dis Colon Rectum 2009 52 11 1869 76 19966635 10.1007/DCR.0b013e3181b35c06

[B33] Zalcman M Sy M Donckier V Closset J Gansbeke DV Helical CT signs in the diagnosis of intestinal ischemia in small-bowel obstruction AJR Am J Roentgenol 2000 175 6 1601 7 11090385 10.2214/ajr.175.6.1751601

[B34] Choi HK Chu KW Law WL Therapeutic value of gastrografin in adhesive small bowel obstruction after unsuccessful conservative treatment: a prospective randomized trial Ann Surg 2002 236 1 6 10.1097/00000658-200207000-00002 12131078 PMC1422541

[B35] Di Saverio S Catena F Ansaloni L Gavioli M Valentino M Pinna AD Water-soluble contrast medium (gastrografin) value in adhesive small intestine obstruction (ASIO): a prospective, randomized, controlled, clinical trial World J Surg 2008 32 10 2293 304 10.1007/s00268-008-9694-6 18688562

[B36] Barkan H Webster S Ozeran S "Factors predicting the recurrence of adhesive small-bowel obstruction" Am J Surg 1995 70 361 365 10.1016/S0002-9610(99)80304-3 7573729

[B37] Foster NM McGory ML Zingmond DS Ko CY Small bowel obstruction: a population-based appraisal J Am Coll Surg 2006 203 170 176 10.1016/j.jamcollsurg.2006.04.020 16864029

[B38] Cox MR Gunn IF Eastman MC Hunt RF Heinz AW The safety and duration of non-operative treatment for adhesive small bowel obstruction Aust N Z J Surg 1993 63 5 367 71 10.1111/j.1445-2197.1993.tb00404.x 8481137

[B39] Fleshner PR Siegman MG Slater GI Brolin RE Chandler JC Aufses AH Jr A prospective, randomized trial of short versus long tubes in adhesive small-bowel obstruction Am J Surg 1995 170 4 366 70 10.1016/S0002-9610(99)80305-5 7573730

[B40] Moran BJ Adhesion-related small bowel obstruction Colorectal Dis 2007 9 Suppl 2 39 44 10.1111/j.1463-1318.2007.01347.x 17824969

[B41] Fevang BT Jensen D Svanes K Viste A Early operation or conservative management of patients with small bowel obstruction? Eur J Surg 2002 168 8-9 475 81 10.1080/110241502321116488 12549688

[B42] Williams SB Greenspon J Young HA Orkin BA Small bowel obstruction: conservative vs. surgical management Dis Colon Rectum 2005 48 6 1140 6 10.1007/s10350-004-0882-7 15906139

[B43] Abbas S Bissett IP Parry BR Oral water soluble contrast for the management of adhesive small bowel obstruction Cochrane Database Syst Rev 2007 18 3 CD004651 10.1002/14651858.CD004651.pub3 PMC6465054 17636770

[B44] Abbas SM Bissett IP Parry BR Meta-analysis of oral water-soluble contrast agent in the management of adhesive small bowel obstruction Br J Surg 2007 94 4 404 11 10.1002/bjs.5775 17380561

[B45] Branco BC Barmparas G Schnüriger B Inaba K Chan LS Demetriades D Systematic review and meta-analysis of the diagnostic and therapeutic role of water-soluble contrast agent in adhesive small bowel obstruction Br J Surg 2010 97 4 470 8 10.1002/bjs.7019 20205228

[B46] Diaz JJ Jr Bokhari F Mowery NT Acosta JA Block EF Bromberg WJ Collier BR Cullinane DC Dwyer KM Griffen MM Mayberry JC Jerome R Guidelines for management of small bowel obstruction J Trauma 2008 64 6 1651 64 10.1097/TA.0b013e31816f709e 18545135

[B47] Sakakibara T Harada A Yaguchi T Koike M Fujiwara M Kodera Y Nakao A The indicator for surgery in adhesive small bowel obstruction patient managed with long tube Hepatogastroenterology 2007 54 75 787 90 17591063

[B48] Komatsu Issei Tokuda Yasuharu Shimada Gen Jacobs Joshua L Hisashi Onodera Development of a simple model for predicting need for surgery in patients who initially undergo conservative management for adhesive small bowel The American Journal of Surgery August 2010 200 2 215 223 10.1016/j.amjsurg.2009.07.045 20591400

[B49] Landercasper J Cogbill TH Merry WH Stolee RT Strutt PJ "Long-term outcome after hospitalization for small-bowel obstruction" Arch Surg 1993 128 765 770 8317958 10.1001/archsurg.1993.01420190059008

[B50] Meagher AP Moller C Hoffmann DC "Non-operative treatment of small bowel obstruction following appendicectomy or operation on the ovary or tube" Br J Surg 1993 80 1310 1311 10.1002/bjs.1800801030 8242308

[B51] Schwenter F Poletti PA Platon A Perneger T Morel P Gervaz P Clinicoradiological score for predicting the risk of strangulated small bowel obstruction Br J Surg 2010 97 7 1119 25 10.1002/bjs.7037 20632281

[B52] Zielinski MD Eiken PW Bannon MP Heller SF Lohse CM Huebner M Sarr MG Small bowel obstruction-who needs an operation? A multivariate prediction model World J Surg 2010 34 5 910 9 10.1007/s00268-010-0479-3 20217412 PMC4882094

[B53] Tanaka S Yamamoto T Kubota D Matsuyama M Uenishi T Kubo S Ono K Predictive factors for surgical indication in adhesive small bowel obstruction Am J Surg 2008 196 1 23 7 10.1016/j.amjsurg.2007.05.048 18367141

[B54] Trésallet C Lebreton N Royer B Leyre P Godiris-Petit G Menegaux F Improving the management of acute adhesive small bowel obstruction with CT-scan and water-soluble contrast medium: a prospective study Dis Colon Rectum 2009 52 11 1869 76 19966635 10.1007/DCR.0b013e3181b35c06

[B55] Abbas S Bissett IP Parry BR Oral water soluble contrast for the management of adhesive small bowel obstruction Cochrane Database Syst Rev 2007 18 3 CD004651 10.1002/14651858.CD004651.pub3 PMC6465054 17636770

[B56] Abbas SM Bissett IP Parry BR Meta-analysis of oral water-soluble contrast agent in the management of adhesive small bowel obstruction Br J Surg 2007 94 4 404 11 10.1002/bjs.5775 17380561

[B57] Branco BC Barmparas G Schnüriger B Inaba K Chan LS Demetriades D Systematic review and meta-analysis of the diagnostic and therapeutic role of water-soluble contrast agent in adhesive small bowel obstruction Br J Surg 2010 97 4 470 8 10.1002/bjs.7019 20205228

[B58] Diaz JJ Jr Bokhari F Mowery NT Acosta JA Block EF Bromberg WJ Collier BR Cullinane DC Dwyer KM Griffen MM Mayberry JC Jerome R Guidelines for management of small bowel obstruction J Trauma 2008 64 6 1651 64 10.1097/TA.0b013e31816f709e 18545135

[B59] Fleshner PR Siegman MG Slater GI Brolin RE Chandler JC Aufses AH Jr A prospective, randomized trial of short versus long tubes in adhesive small-bowel obstruction Am J Surg 1995 170 4 366 70 10.1016/S0002-9610(99)80305-5 7573730

[B60] Diaz JJ Jr Bokhari F Mowery NT Acosta JA Block EF Bromberg WJ Collier BR Cullinane DC Dwyer KM Griffen MM Mayberry JC Jerome R Guidelines for management of small bowel obstruction J Trauma 2008 64 6 1651 64 10.1097/TA.0b013e31816f709e 18545135

[B61] Sakakibara T Harada A Yaguchi T Koike M Fujiwara M Kodera Y Nakao A The indicator for surgery in adhesive small bowel obstruction patient managed with long tube Hepatogastroenterology 2007 54 75 787 90 17591063

[B62] Assalia A Schein M Kopelman D Hirshberg A Hashmonai M Therapeutic effect of oral Gastrografin in adhesive, partial small-bowel obstruction: a prospective randomized trial Surgery 1994 115 4 433 7 8165534

[B63] Assalia A Kopelman D Bahous H Klein Y Hashmonai M Gastrografin for mechanical partial, small bowel obstruction due to adhesions Harefuah 1997 132 9 629 33 9225576

[B64] Choi HK Chu KW Law WL Therapeutic value of gastrografin in adhesive small bowel obstruction after unsuccessful conservative treatment: a prospective randomized trial Ann Surg 2002 236 1 1 6 10.1097/00000658-200207000-00002 12131078 PMC1422541

[B65] Choi HK Law WL Ho JW Chu KW Value of gastrografin in adhesive small bowel obstruction after unsuccessful conservative treatment: a prospective evaluation World J Gastroenterol 2005 11 24 3742 5 15968731 10.3748/wjg.v11.i24.3742 PMC4316027

[B66] Biondo S Parés D Mora L Martí Ragué J Kreisler E Jaurrieta E Randomized clinical study of Gastrografin administration in patients with adhesive small bowel obstruction J Surg 2003 90 5 542 6 10.1002/bjs.4150 12734858

[B67] Burge J Abbas SM Roadley G Donald J Connolly A Bissett IP Hill AG Randomized controlled trial of Gastrografin in adhesive small bowel obstruction ANZ J Surg 2005 75 8 672 4 10.1111/j.1445-2197.2005.03491.x 16076330

[B68] Di Saverio S Catena F Ansaloni L Gavioli M Valentino M Pinna AD Water-soluble contrast medium (gastrografin) value in adhesive small intestine obstruction (ASIO): a prospective, randomized, controlled, clinical trial World J Surg 2008 32 10 2293 304 10.1007/s00268-008-9694-6 18688562

[B69] Abbas SM Bissett IP Parry BR Meta-analysis of oral water-soluble contrast agent in the management of adhesive small bowel obstruction Br J Surg 2007 94 4 404 11 10.1002/bjs.5775 17380561

[B70] Abbas S Bissett IP Parry BR Oral water soluble contrast for the management of adhesive small bowel obstruction Cochrane Database Syst Rev 2007 18 3 CD004651 10.1002/14651858.CD004651.pub3 PMC6465054 17636770

[B71] Branco BC Barmparas G Schnüriger B Inaba K Chan LS Demetriades D Systematic review and meta-analysis of the diagnostic and therapeutic role of water-soluble contrast agent in adhesive small bowel obstruction Br J Surg 2010 97 4 470 8 10.1002/bjs.7019 20205228

[B72] Diaz JJ Jr Bokhari F Mowery NT Acosta JA Block EF Bromberg WJ Collier BR Cullinane DC Dwyer KM Griffen MM Mayberry JC Jerome R Guidelines for management of small bowel obstruction J Trauma 2008 64 6 1651 64 10.1097/TA.0b013e31816f709e 18545135

[B73] Chen SC Yen ZS Lee CC Liu YP Chen WJ Lai HS Lin FY Chen WJ Nonsurgical management of partial adhesive small-bowel obstruction with oral therapy: a randomized controlled trial CMAJ 2005 173 10 1165 9 16275967 10.1503/cmaj.1041315 PMC1277043

[B74] Ambiru S Furuyama N Kimura F Shimizu H Yoshidome H Miyazaki M Ochiai T Effect of hyperbaric oxygen therapy on patients with adhesive intestinal obstruction associated with abdominal surgery who have failed to respond to more than 7 days of conservative treatment Hepatogastroenterology 2008 55 82-83 491 5 18613394

[B75] Shih Shou-Chuan Jeng Kuo-Shyang Shee-Chan Lin Adhesive small bowel obstruction: How long can patients tolerate conservative treatment? World J Gastroenterol 2003 9 3 603 605 12632527 10.3748/wjg.v9.i3.603 PMC4621591

[B76] Cox MR Gunn IF Eastman MC Hunt RF Heinz AW The safety and duration of non-operative treatment for adhesive small bowel obstruction Aust N Z J Surg 1993 63 5 367 71 10.1111/j.1445-2197.1993.tb00404.x 8481137

[B77] Fleshner PR Siegman MG Slater GI Brolin RE Chandler JC Aufses AH Jr A prospective, randomized trial of short versus long tubes in adhesive small-bowel obstruction Am J Surg 1995 170 4 366 70 10.1016/S0002-9610(99)80305-5 7573730

[B78] Gowen GF Long tube decompression is successful in 90% of patients with adhesive small bowel obstruction Am J Surg 2003 185 6 512 5 10.1016/S0002-9610(03)00074-6 12781876

[B79] Tanaka S Yamamoto T Kubota D Matsuyama M Uenishi T Kubo S Ono K Predictive factors for surgical indication in adhesive small bowel obstruction Am J Surg 2008 196 1 23 7 10.1016/j.amjsurg.2007.05.048 18367141

[B80] Sakakibara T Harada A Yaguchi T Koike M Kodera Y Nakao A The indicator for surgery in adhesive small bowel obstruction patient managed with long tube Hepatogastroenterology 2007 54 75 787 90 17591063

[B81] Diaz JJ Jr Bokhari F Mowery NT Acosta JA Block EF Bromberg WJ Collier BR Cullinane DC Dwyer KM Griffen MM Mayberry JC Jerome R Guidelines for management of small bowel obstruction J Trauma 2008 64 6 1651 64 10.1097/TA.0b013e31816f709e 18545135

[B82] Foster NM McGory ML Zingmond DS Ko CY Small bowel obstruction: a population-based appraisal J Am Coll Surg 2006 203 170 176 10.1016/j.jamcollsurg.2006.04.020 16864029

[B83] Duron JJ Silva NJ du Montcel ST Berger A Muscari F Hennet H Veyrieres M Hay JM Adhesive postoperative small bowel obstruction: incidence and risk factors of recurrence after surgical treatment: a multicenter prospective study Ann Surg 2006 244 5 750 7 10.1097/01.sla.0000225097.60142.68 17060768 PMC1856591

[B84] Miller G Boman J Shrier I Gordon PH Natural history of patients with adhesive small bowel obstruction Br J Surg 2000 87 9 1240 7 10.1046/j.1365-2168.2000.01530.x 10971435

[B85] Sakakibara T Harada A Yaguchi T Koike M Fujiwara M Nakao A The indicator for surgery in adhesive small bowel obstruction patient managed with long tube Hepatogastroenterology 2007 54 75 787 90 17591063

[B86] Sakakibara T Harada A Ishikawa Komatsu Yaguchi Kodera Nakao A Parameter predicting the recurrence of adhesive small bowel obstruction in patients managed with a long tube World J Surg 2007 31 1 80 5 10.1007/s00268-006-0158-6 17180476

[B87] Fevang BT Fevang J Lie SA Søreide O Svanes K Viste A Long-term prognosis after operation for adhesive small bowel obstruction Ann Surg 2004 240 2 193 201 10.1097/01.sla.0000132988.50122.de 15273540 PMC1356393

[B88] Williams SB Greenspon J Young HA Orkin BA Small bowel obstruction: conservative vs. surgical management Dis Colon Rectum 2005 48 6 1140 6 10.1007/s10350-004-0882-7 15906139

[B89] Di Saverio S Catena F Ansaloni L Gavioli M Valentino M Pinna AD Water-soluble contrast medium (gastrografin) value in adhesive small intestine obstruction (ASIO): a prospective, randomized, controlled, clinical trial World J Surg 2008 32 10 2293 304 10.1007/s00268-008-9694-6 18688562

[B90] Scott-Coombes DM Vipond MN Thompson JM "General surgeons attitudes to the treatment and prevention of abdominal adhesions" Ann R Coll Surg Engl 1993 75 123 128 8476180 PMC2497791

[B91] Brill AI Nezhat F Nezhat CH Nezhat C The incidence of adhesion after prior laparotomy: a laparoscopic appraisal Obstet Gynecol 1995 85 6 269 72 10.1016/0029-7844(94)00352-E 7824244

[B92] Levrant SG Bieber E Barnes R Risk of anterior abdominal wall adhesions increases with number and type of previous laparotomy J Am Assoc Gynecol Laparosc 1994 1 4 S19 10.1016/S1074-3804(05)80928-4 9073706

[B93] Van Der Krabben AA Dijkstra FR Nieuwenhuijzen M Morbidity and mortality of inadvertent enterotomy during adhesiolysis Br J Surg 2000 87 467 71 10.1046/j.1365-2168.2000.01394.x 10759744

[B94] Fazio VW Reduction in adhesive small-bowel obstruction by Seprafilm adhesion barrier after intestinal resection Dis Colon Rectum 2006 49 1 1 11 10.1007/s10350-005-0268-5 16320005

[B95] Van Der Krabben AA Dijkstra FR Nieuwenhuijzen M Morbidity and mortality of inadvertent enterotomy during adhesiolysis Br J Surg 2000 87 467 71 10.1046/j.1365-2168.2000.01394.x 10759744

[B96] Landercasper J Cogbill TH Merry WH Long-term outcome after hospitalization for small-bowel obstruction Arch Surg 1993 128 765 770 8317958 10.1001/archsurg.1993.01420190059008

[B97] Tittel A Treutner KH Titkova S Comparison of adhesion reformation after laparoscopic and conventional adhesiolysis in an animal model. Langenbeck's Arch Surg 2001 386 141 145 10.1007/s004230000190 11374047

[B98] Gamal EM Metzger P Szabo G The influence of intraoperative complications on adhesion formation during laparoscopic and conventional cholecystectomy in an animal model Surg Endosc 2001 15 873 7 10.1007/s004640000358 11443424

[B99] Gadallah MF Torres-Rivera C Ramdeen G Myrick S Habashi S Andrews G Relationship between intraperitoneal bleeding, adhesions, and peritoneal dialysis catheter failure: a method of prevention Adv Perit Dial 2001 17 127 9 11510259

[B100] Nagle A Ujiki M Denham W Murayama K Laparoscopic adhesiolysis for small bowel obstruction Am J Surg 2004 187 4 464 70 10.1016/j.amjsurg.2003.12.036 15041492

[B101] Swank DJ Swank-Bordewijk SC Hop WC van Erp WF Janssen IM Bonjer HJ Jeekel J Laparoscopic adhesiolysis in patients with chronic abdominal pain: a blinded randomised controlled multi-centre trial Lancet 2003 361 9365 1247 51 10.1016/S0140-6736(03)12979-0 12699951

[B102] Cirocchi R Abraha I Farinella E Montedori A Sciannameo F Laparoscopic versus open surgery in small bowel obstruction Cochrane Database Syst Rev 2010 17 2 CD007511 Review 10.1002/14651858.CD007511.pub2 PMC7202250 20166096

[B103] Ray NF Denton WG Thamer M Henderson SC Perry S Abdominal adhesiolysis: inpatient care and expenditures in the United States in 1994 J Am Coll Surg 1998 186 1 9 10.1016/S1072-7515(97)00127-0 9449594

[B104] Suter M Zermatten P Hakic N Laparoscopic management of mechanical small bowel obstruction: are there predictors of success or failure? Surg Endosc 2000 14 478 484 10.1007/s004640000104 10858476

[B105] León EL Metzger A Tsiotos GG Laparoscopic management of small bowel obstruction: indications and outcomes J Gastrointest Surg 1998 2 132 140 9834408 10.1016/s1091-255x(98)80003-6

[B106] Navez B Arimont JM Guit P Laparoscopic approach in acute small bowel obstruction. A review of 68 patients Hepatogastroenterology 1998 45 2146 2150 9951882

[B107] Suter M Zermatten P Hakic N Laparoscopic management of mechanical small bowel obstruction: are there predictors of success or failure? Surg Endosc 2000 14 478 484 10.1007/s004640000104 10858476

[B108] Pekmezci S Altinli E Saribeyoglu K Enteroclysis-guided laparoscopic adhesiolysis in recurrent adhesive small bowel obstructions Surg Laparosc Endosc Percutan Tech 2001 12 165 170 10.1097/00129689-200206000-00005 12080256

[B109] Leon EL Metzger A Tsiotos GG Schlinkert RT Sarr MG Laparoscopic management of acute small bowel obstruction: indications and outcome J Gastrointest Surg 1998 2 132 40 10.1016/S1091-255X(98)80003-6 9834408

[B110] Wang Q Hu ZQ Wang WJ Zhang J Wang Y Ruan CP Laparoscopic management of recurrent adhesive small-bowel obstruction: Long-term follow-up Surg Today 2009 39 6 493 9 10.1007/s00595-008-3906-4 19468805

[B111] Navez B Arimont JM Guit P Laparoscopic approach in acute small bowel obstruction. A review of 68 patients Hepatogastroenterology 1998 45 2146 2150 9951882

[B112] Van Goor H Consequences and complications of peritoneal adhesions Colorectal Dis 2007 9 Suppl 2 25 34 10.1111/j.1463-1318.2007.01358.x 17824967

[B113] Sato Y Ido K Kumagai M Laparoscopic adhesiolysis for recurrent small bowel obstruction: long-term follow-up Gastrointest Endosc 2001 54 476 479 10.1067/mge.2001.117760 11577310

[B114] Chosidow D Johanet H Montario T Laparoscopy for acute small-bowel obstruction secondary to adhesions J Laparoendosc Adv Surg Tech 2000 10 155 159 10.1089/lap.2000.10.155 10883993

[B115] Sato Y Ido K Kumagai M Laparoscopic adhesiolysis for recurrent small bowel obstruction: long-term follow-up Gastrointest Endosc 2001 54 476 479 10.1067/mge.2001.117760 11577310

[B116] Farinella E Cirocchi R La Mura F Morelli U Cattorini L Delmonaco P Migliaccio C De Sol AA Cozzaglio L Sciannameo F Feasibility of laparoscopy for small bowel obstruction World J Emerg Surg 2009 4 3 10.1186/1749-7922-4-3 19152695 PMC2639545

[B117] Wullstein C Gross E Laparoscopic compared with conventional treatment of acute adhesive small bowel obstruction Br J Surg 2003 90 1147 51 10.1002/bjs.4177 12945085

[B118] Khaikin M Schneidereit N Cera S Sands D Efron J Weiss G Nogueras JJ Vernava AM Wexner SD Laparoscopic vs. open surgery for acute adhesive small-bowel obstruction: patient' outcome and cost-effextiveness Surg Endosc 2007 21 742 746 10.1007/s00464-007-9212-1 17332956

[B119] Franklin ME Gonzales JJ Miter DB Glass JL Paulson D Laparoscopic diagnosis and treatment of intestinal obstruction Surg Endosc 2004 18 26 30 10.1007/s00464-003-8804-7 14625729

[B120] Franklin ME Dorman JP Pharand D Laparoscopic surgery in acute small obstruction Surg Laparosc Endosc 1994 4 289 96 10.1097/00019509-199410000-00057 7952440

[B121] Peschaud F Alves A Berdah S Kianmanesh R Lurent C Ma Brut JY Mariette C Meurette G Pirro N Veryrie N Slim K Indicazioni alla laparoscopia in chirurgia generale e digestiva J Chir 2006 6 65 79

[B122] Levard H Boudet MJ Msika S Molkhou JM Hay JM La Borde Y Gilet M Fingerhut A French Association for Surgical Research: Laparoscopic treatment of acute small bowel obstruction: a multicentre retrospective study ANZ J Surg 2001 71 641 46 10.1046/j.0004-8682.2001.02222.x 11736822

[B123] Leon EL Metzger A Tsiotos GG Schlinkert RT Sarr MG Laparoscopic management of acute small bowel obstruction: indications and outcome J Gastrointest Surg 1998 2 132 40 10.1016/S1091-255X(98)80003-6 9834408

[B124] Franklin ME Gonzales JJ Miter DB Glass JL Paulson D Laparoscopic diagnosis and treatment of intestinal obstruction Surg Endosc 2004 18 26 30 10.1007/s00464-003-8804-7 14625729

[B125] Levard H Boudet MJ Msika S Laparoscopic treatment of acute small bowel obstruction: a multicentre retrospective study A N Z J Surg 2001 71 641 646 10.1046/j.0004-8682.2001.02222.x 11736822

[B126] Duron JJ du Montcel ST Berger A Muscari F Hennet H Veyrieres M Hay JM French Federation for Surgical Research. Prevalence and risk factors of mortality and morbidity after operation for adhesive postoperative small bowel obstruction Am J Surg 2008 195 6 726 34 10.1016/j.amjsurg.2007.04.019 18367136

[B127] Duron JJ Silva NJ du Montcel ST Berger A Muscari F Hennet H Veyrieres M Hay JM Adhesive postoperative small bowel obstruction: incidence and risk factors of recurrence after surgical treatment: a multicenter prospective study Ann Surg 2006 244 5 750 7 10.1097/01.sla.0000225097.60142.68 17060768 PMC1856591

[B128] Mancini GJ Petroski GF Lin WC Sporn E Miedema BW Thaler K Nationwide impact of laparoscopic lysis of adhesions in the management of intestinal obstruction in the US J Am Coll Surg 2008 207 4 520 6 10.1016/j.jamcollsurg.2008.04.026 18926453

[B129] Szomstein S Lo Menzo E Simpfendorfer C Laparoscopic lysis of adhesions World J Surg 2006 30 535 540 10.1007/s00268-005-7778-0 16555020

[B130] Grafen FC Neuhaus V Schöb O Turina M Management of acute small bowel obstruction from intestinal adhesions: indications for laparoscopic surgery in a community teaching hospital Langenbecks Arch Surg 2010 395 1 57 63 10.1007/s00423-009-0490-z 19330347

[B131] Zerey M Sechrist CW Kercher KW Sing RF Matthews BD Heniford BT Laparoscopic management of adhesive small bowel obstruction Am Surg 2007 73 8 773 8 17879683

[B132] Borzellino G Tasselli S Zerman G Pedrazzani C Manzoni G Laparoscopic approach to postoperative adhesive obstruction Surg Endosc 2004 18 4 686 90 10.1007/s00464-003-9106-9 15026903

[B133] Sato Y Ido K Kumagai M Isoda N Hozumi M Nagamine N Ono K Shibusawa H Togashi K Sugano K Laparoscopic adhesiolysis for recurrent small bowel obstruction: long-term follow-up Gastrointest Endosc 2001 54 4 476 9 10.1067/mge.2001.117760 11577310

[B134] Tsumura H Ichikawa T Murakami Y Sueda T Laparoscopic adhesiolysis for recurrent postoperative small bowel obstruction Hepatogastroenterology 2004 51 58 1058 61 15239246

[B135] Léon EL Metzger A Tsiotos GG Schlinkert RT Sarr MG Laparoscopic management of small bowel obstruction: indications and outcome J Gastrointest Surg 1998 2 2 132 40 9834408 10.1016/s1091-255x(98)80003-6

[B136] Ghosheh B Salameh JR Laparoscopic approach to acute small bowel obstruction: review of 1061 cases Surg Endosc 2007 21 1945 1949 10.1007/s00464-007-9575-3 17879114

[B137] Nagle A Ujiki M Denham W Murayama K Laparoscopic adhesiolysis for small bowel obstruction Am J Surg 2004 187 4 464 70 10.1016/j.amjsurg.2003.12.036 15041492

[B138] Strickland P Lourie DJ Suddleson EA Blitz JB Stain SC Is laparoscopy safe and effective for treatment of acute small-bowel obstruction? Surg Endosc 1999 13 7 695 8 10.1007/s004649901075 10384077

[B139] Levard H Boudet MJ Msika S Molkhou JM Hay JM Laborde Y French Association for Surgical Research. Laparoscopic treatment of acute small bowel obstruction: a multicentre retrospective study Aust N Z J Surg 2001 71 641 6 10.1046/j.0004-8682.2001.02222.x 11736822

[B140] Toouli J, Gossot D, Hunter JG Duh QY Small bowel obstruction Endosurgery Churchill Livingstone 1998 New York 425 431

[B141] Barmparas G Branco BC Schnüriger B Lam L Inaba K Demetriades D The incidence and risk factors of post-laparotomy adhesive small bowel obstruction J Gastrointest Surg 2010 14 10 1619 28 10.1007/s11605-010-1189-8 20352368

[B142] Fevang BT Fevang J Lie SA Søreide O Svanes K Viste A Long-term prognosis after operation for adhesive small bowel obstruction Ann Surg 2004 240 2 193 201 10.1097/01.sla.0000132988.50122.de 15273540 PMC1356393

[B143] Duron JJ Silva NJ du Montcel ST Berger A Muscari F Hennet H Veyrieres M Hay JM Adhesive postoperative small bowel obstruction: incidence and risk factors of recurrence after surgical treatment: a multicenter prospective study Ann Surg 2006 244 5 750 7 10.1097/01.sla.0000225097.60142.68 17060768 PMC1856591

[B144] Hackethal A Sick C Brueggmann D Tchartchian G Wallwiener M Muenstedt K Tinneberg HR Awareness and perception of intra-abdominal adhesions and related consequences: survey of gynaecologists in German hospitals Eur J Obstet Gynecol Reprod Biol 2010 150 2 180 9 10.1016/j.ejogrb.2010.02.017 20236750

[B145] Schreinemacher MH Ten Broek RP Bakkum EA van Goor H Bouvy ND Adhesion Awareness: A National Survey of Surgeons World J Surg 2010 34 12 2805 2812 10.1007/s00268-010-0778-8 20814678 PMC2982960

[B146] Schnüriger Beat Barmparas Galinos Branco Bernardino C Lustenberger Thomas Inaba Kenji Demetrios Demetriades Prevention of postoperative peritoneal adhesions: a review of the literature The American Journal of Surgery10.1016/j.amjsurg.2010.02.008 20817145

[B147] Soybir GR Koksoy F Polat C The effects of sterile or infected bile and dropped gallstones in abdominal adhesions and abscess formation Surg Endosc 1997 11 711 713 10.1007/s004649900433 9214316

[B148] van den Tol P Haverlag R van Rossen ME Glove powder promotes adhesion formation and facilitates tumour cell adhesion and growth Br J Surg 2001 88 1258 1263 10.1046/j.0007-1323.2001.01846.x 11531877

[B149] Cooke A Hamilton DG The significance of starch powder contamination in the aetiology of peritoneal adhesions Br J Surg 1977 64 410 412 10.1002/bjs.1800640610 871614

[B150] Barmparas G Branco BC Schnüriger B Lam L Inaba K Demetriades D The incidence and risk factors of post-laparotomy adhesive small bowel obstruction J Gastrointest Surg 2010 14 10 1619 28 10.1007/s11605-010-1189-8 20352368

[B151] Fevang BT Fevang J Lie SA Søreide O Svanes K Viste A Long-term prognosis after operation for adhesive small bowel obstruction Ann Surg 2004 240 2 193 201 10.1097/01.sla.0000132988.50122.de 15273540 PMC1356393

[B152] Barmparas G Branco BC Schnüriger B Lam L Inaba K Demetriades D The incidence and risk factors of post-laparotomy adhesive small bowel obstruction J Gastrointest Surg 2010 14 10 1619 28 10.1007/s11605-010-1189-8 20352368

[B153] Schnüriger Beat Barmparas Galinos Branco Bernardino C Lustenberger Thomas Inaba Kenji Demetrios Demetriades Prevention of postoperative peritoneal adhesions: a review of the literature The American Journal of Surgery10.1016/j.amjsurg.2010.02.008 20817145

[B154] Malvasi A Tinelli A Farine D Effects of visceral peritoneal closure on scar formation at cesarean delivery Int J Gynecol Obstet 2009 105 131 135 10.1016/j.ijgo.2008.12.019 19232595

[B155] Beck DE Cohen Z Fleshman JW A prospective, randomized, multicenter, controlled study of the safety of Seprafilm adhesion barrier in abdominopelvic surgery of the intestine Dis Colon Rectum 2003 46 1310 1319 10.1007/s10350-004-6739-2 14530667

[B156] Becker M Dayton MT Fazio VW Prevention of postoperative abdominal adhesions by a sodium hyaluronate-based bioresorbable membrane: a prospective, randomized, double-blind multicenter study J Am Coll Surg 1996 183 297 306 8843257

[B157] Vrijland WW Tseng LN Eijkman HJ Fewer intraperitoneal adhesions with use of hyaluronic acid-carboxymethylcellulose membrane: a randomized clinical trial Ann Surg 2002 235 193 199 10.1097/00000658-200202000-00006 11807358 PMC1422414

[B158] Cohen Z Senagore AJ Dayton MT Prevention of postoperative abdominal adhesions by a novel, glycerol/sodium hyaluronate/carboxymethylcellulose-based bioresorbable membrane: a prospective, randomized, evaluator-blinded multicenter study Dis Colon Rectum 2005 48 1130 1139 10.1007/s10350-004-0954-8 15868230

[B159] Kumar S Wong PF Leaper DJ Intra-peritoneal prophylactic agents for preventing adhesions and adhesive intestinal obstruction after non-gynaecological abdominal surgery Cochrane Database Syst Rev 2009 1 CD005080 19160246 10.1002/14651858.CD005080.pub2 PMC13369267

[B160] Fazio VW Cohen Z Fleshman JW Reduction in adhesive small-bowel obstruction by Seprafilm adhesion barrier after intestinal resection Dis Colon Rectum 2006 49 1 11 10.1007/s10350-005-0268-5 16320005

[B161] Kudo FA Nishibe T Miyazaki K Use of bioresorbable membrane to prevent postoperative small bowel obstruction in transabdominal aortic aneurysm surgery Surg Today 2004 34 648 651 15290392 10.1007/s00595-004-2792-7

[B162] Vrijland WW Tseng LN Eijkman HJ Hop WC Jakimowicz JJ Leguit P Stassen LP Swank DJ Haverlag R Bonjer HJ Jeekel H Fewer intraperitoneal adhesions with use of hyaluronic acid-carboxymethylcellulose membrane: a randomized clinical trial Ann Surg 2002 235 2 193 9 10.1097/00000658-200202000-00006 11807358 PMC1422414

[B163] Zeng Q Yu Z You J Zhang Q Efficacy and safety of Seprafilm for preventing postoperative abdominal adhesion: systematic review and meta-analysis World J Surg 2007 31 11 2125 31 10.1007/s00268-007-9242-9 17899250

[B164] Kumar S Wong PF Leaper DJ Intra-peritoneal prophylactic agents for preventing adhesions and adhesive intestinal obstruction after non-gynaecological abdominal surgery Cochrane Database Syst Rev 2009 21 1 CD005080 10.1002/14651858.CD005080.pub2 PMC13369267 19160246

[B165] Prevention of postsurgical adhesions by INTERCEED(TC7), an absorbable adhesion barrier: a prospective randomized multicenter clinical study INTERCEED(TC7) Adhesion Barrier Study Group Fertil Steril 1989 51 6 933 8 2524407

[B166] Saravelos H Li TC Post-operative adhesions after laparoscopic electrosurgical treatment for polycystic ovarian syndrome with the application of Interceed to one ovary: a prospective randomized controlled study Hum Reprod 1996 11 5 992 7 8671376 10.1093/oxfordjournals.humrep.a019337

[B167] Azziz R Microsurgery alone or with INTERCEED absorbable adhesion barrier for pelvic sidewall adhesion re-formation: The INTERCEED (TC7) Adhesion Barrier Study Group. II Surg Gynecol Obstet 1993 177 135 139 8342092

[B168] The efficacy of Interceed (TC7)* for prevention of reformation of postoperative adhesions on ovaries, fallopian tubes, and fimbriae in microsurgical operations for fertility: a multicenter study: Nordic Adhesion Prevention Study Group Fertil Steril 1995 63 709 714 7890052

[B169] Wiseman DM Trout JR Franklin RR Metaanalysis of the safety and efficacy of an adhesion barrier (Interceed TC7) in laparotomy J Reprod Med 1999 44 325 331 10319300

[B170] Ahmad G Duffy JM Farquhar C Barrier agents for adhesion prevention after gynaecological surgery Cochrane Database Syst Rev 2008 16 CD000475 10.1002/14651858.CD000475.pub2 18425865

[B171] Montz FJ Monk BJ Lacy SM The Gore-Tex surgical membrane: effectiveness as a barrier to inhibit postradical pelvic surgery adhesions in a porcine model Gynecol Oncol 1992 45 290 293 10.1016/0090-8258(92)90306-4 1612506

[B172] Bhardwaj R Parker MC Impact of adhesions in colorectal surgery Colorectal Dis 2007 9 Suppl 2 45 53 10.1111/j.1463-1318.2007.01357.x 17824970

[B173] Ahmad G Duffy JM Farquhar C Vail A Vandekerckhove P Watson A Wiseman D Barrier agents for adhesion prevention after gynaecological surgery Cochrane Database Syst Rev 2008 2 CD000475 10.1002/14651858.CD000475.pub2 18425865

[B174] Metwally M Watson A Lilford R Vandekerckhove P Fluid and pharmacological agents for adhesion prevention after gynaecological surgery Cochrane Database Syst Rev 2006 2 CD001298 16625541 10.1002/14651858.CD001298.pub3

[B175] Brown CB Luciano AA Martin D Adept (icodextrin 4% solution) reduces adhesions after laparoscopic surgery for adhesiolysis: a double-blind, randomized, controlled study Fertil Steril 2007 88 1413 1426 10.1016/j.fertnstert.2006.12.084 17383643

[B176] Kössi J Grönlund S Uotila-Nieminen M Crowe A Knight A Keränen U The effect of 4% icodextrin solution on adhesiolysis surgery time at the Hartmann's reversal: a pilot, multicentre, randomized control trial vs lactated Ringer's solution Colorectal Dis 2009 11 2 168 72 18462234 10.1111/j.1463-1318.2008.01562.x

[B177] Catena F Ansaloni L Lauro A Ercolani G D'Alessandro L Pinna A Prospective controlled randomized trial on prevention of postoperative abdominal adhesions by Icodextrin 4% solution after laparotomic operation for small bowel obstruction caused by adherences [POPA study: Prevention of Postoperative Adhesions on behalf of the World Society of Emergency Surgery] Trials 2008 9 74 10.1186/1745-6215-9-74 19094225 PMC2631497

[B178] Menzies D Pascual MH Walz MK Duron JJ Tonelli F Crowe A Knight A ARIEL Registry. Use of icodextrin 4% solution in the prevention of adhesion formation following general surgery: from the multicentre ARIEL Registry Ann R Coll Surg Engl 2006 88 4 375 82 10.1308/003588406X114730 16834859 PMC1964633

[B179] Johns DA Ferland R Dunn R Initial feasibility study of a sprayable hydrogel adhesion barrier system in patients undergoing laparoscopic ovarian surgery J Am Assoc Gynecol Laparosc 2003 10 334 338 10.1016/S1074-3804(05)60257-5 14567807

[B180] Tang CL Jayne DG Seow-Choen F A randomized controlled trial of .5% ferric hyaluronate gel (Intergel) in the prevention of adhesions following abdominal surgery Ann Surg 2006 243 449 455 10.1097/01.sla.0000207837.71831.a2 16552194 PMC1448972

[B181] Sparnon AL Spitz L Pharmacological manipulation of postoperative intestinal adhesions Aust N Z J Surg 1989 59 725 9 10.1111/j.1445-2197.1989.tb01665.x 2783096

[B182] Fang CC Chou TH Lin GS Yen ZS Lee CC Chen SC Peritoneal infusion with cold saline decreased postoperative intra-abdominal adhesion formation World J Surg 2010 34 4 721 7 10.1007/s00268-009-0378-7 20049434

